# A Multifunctional Molecular Probe for Multimodal Imaging‐Guided Potent Photothermal/Photodynamic Therapy of Endometriosis

**DOI:** 10.1002/advs.202511126

**Published:** 2025-08-28

**Authors:** Qiyu Zhong, Shuguang Yang, Xiao Li, Zhuang Jin, Jianyu Ma, Zhouzhou Liao, Jinbo Li, Bo Li, Xintao Shuai, Shuqin Chen

**Affiliations:** ^1^ Department of Gynecology The Sixth Affiliated Hospital Sun Yat‐sen University Guangzhou 510655 China; ^2^ Biomedical Innovation Center The Sixth Affiliated Hospital Sun Yat‐sen University Guangzhou 510655 China; ^3^ Nanomedicine Research Center The Third Affiliated Hospital of Sun Yat‐sen University Guangzhou 510630 China; ^4^ Department of Orthopaedics Shanghai Key Laboratory for Prevention and Treatment of Bone and Joint Diseases Shanghai Institute of Traumatology and Orthopaedics Ruijin Hospital Shanghai Jiao Tong University School of Medicine 197 Ruijin 2nd Road Shanghai 200025 China; ^5^ Guangzhou Municipal and Guangdong Provincial Key Laboratory of Molecular Target & Clinical Pharmacology the NMPA and State Key Laboratory of Respiratory Disease School of Pharmaceutical Sciences Guangzhou Medical University Guangzhou 511436 China

**Keywords:** endometriosis, multimodal imaging, photodynamic therapy, photothermal therapy, small molecular probe

## Abstract

Multifunctional small‐molecule theranostic agents hold significant clinical potential for non‐invasive endometriosis (EMS) management. Current EMS treatment faces challenges due to imprecise lesion localization and therapy‐associated side effects. Herein, an integrated theranostic probe, cRGDyK‐ICG‐Lys‐DTPA@Gd (cRGD‐ILD), designed for concurrent precise lesion localization and targeted phototherapy in EMS, is developed. This molecular probe integrates three key functional components: the DTPA@Gd complex provides contrast for magnetic resonance imaging, the ICG fragment enables fluorescence imaging and photoacoustic imaging while serving as the phototherapeutic agent, and the cRGD peptide targets integrin *α*_v_ *β*_3_ receptors to facilitate precise accumulation in ectopic endometrial tissue. Specifically, cRGD‐ILD leverages its small size and receptor‐mediated transcytosis to penetrate neovascularized tissue and target ectopic endometrial cells. Under 808 nm laser irradiation, the ICG moiety generates reactive oxygen species and heat, exerting combined photodynamic and photothermal therapeutic effects that effectively suppress lesion progression in both autografted EMS rats and xenografted nude mice. Notably, composed of clinically available and FDA‐approved constituents, the probe demonstrates favorable biocompatibility and biosafety for in vivo applications. Overall, this well‐designed molecular probe enables precise multimodal imaging guidance and localized phototherapy for EMS.

## Graphical abstract

The cRGD‐ILD molecular probe integrates clinically available components: a cRGD peptide (integrin *α*_v_ *β*_3_ targeting), ICG (for fluorescence/photoacoustic imaging and phototherapy), and Gd‐DTPA (MRI contrast). Utilizing small size and receptor targeting, it achieves selective lesion accumulation. Under 808 nm irradiation, ICG generates heat and ROS for combined photothermal/photodynamic therapy, validated in rodent endometriosis models, enabling precise imaging‐guided phototherapy.

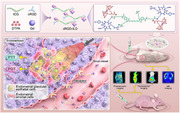

## 1 Introduction

Endometriosis (EMS) represents a severe gynecological disorder wherein endometrial tissue proliferates outside the confines of the uterine cavity, significantly impairing patients' quality of life and mental well‐being. With a prevalence of 10% and rising incidence, EMS serves as a prominent underlying factor of chronic pelvic pain, dysmenorrhea, and infertility.^[^ ^1^ ^]^ Notably, 20%–50% of infertile women^[^ ^2^ ^]^ and 71%–87% of those with chronic pelvic pain^[^ ^3^ ^]^ are diagnosed with EMS. Current management strategies, primarily surgical excision and pharmacotherapy, face significant limitations.^[^ ^4^ ^]^ Surgical intervention is hindered by the diffuse, multifocal, and often occult nature of lesions, which complicates complete resection and risks ovarian reserve depletion, iatrogenic injury, and recurrence.^[^ ^5^ ^]^ Pharmacological approaches, though effective in symptom management, are frequently limited by systemic side effects (e.g., hypoestrogenic symptoms, ovulatory suppression) and dose‐limiting toxicities that preclude long‐term use.^[^ ^6^ ^]^ Consequently, improving lesion detection accuracy and developing targeted therapies with reduced off‐target effects remain critical unmet needs in EMS management.

Accurate lesion delineation remains pivotal for effective EMS management, yet existing imaging modalities demonstrate variable performance. Computed tomography provides rapid anatomical assessment but lacks sufficient soft‐tissue contrast resolution to reliably differentiate endometriotic lesions from fibroids or early pelvic malignancies.^[^ ^7^ ^]^ Transvaginal ultrasonography is widely accessible and radiation‐free, yet its sensitivity is often reduced for non‐cystic lesions due to insufficient acoustic impedance differences between ectopic implants and surrounding tissue.^[^ ^8^ ^]^ Magnetic resonance imaging (MRI) provides superior soft‐tissue contrast and multiplanar capability, making it particularly valuable for complex extrapelvic lesions and surgical planning.^[^ ^9^ ^]^ Gadolinium‐based contrast agents enhance MRI sensitivity by shortening *T*_1_ relaxation times,^[^ ^10^ ^]^ though their non‐specific biodistribution can compromise diagnostic selectivity and raise safety concerns in renal impairment.^[^ ^11^ ^]^ Emerging techniques like photoacoustic imaging (PAI) leverage near‐infrared (NIR)‐induced thermoelastic expansion to generate ultrasound signals,^[^ ^12^ ^]^ permitting real‐time visualization of metabolic and vascular parameters within endometriotic foci.^[^ ^13^ ^]^ Crucially, multimodal fusion of PAI with MRI synergistically contextualizes molecular‐functional data within high‐resolution anatomical frameworks,^[^ ^14^ ^]^ significantly enhancing diagnostic precision.^[^ ^15^ ^]^

Concurrently, precision medicine approaches leveraging molecular targeting have gained traction, particularly through integrin‐mediated delivery systems.^[^ ^16^ ^]^ Integrin receptors, particularly *α*_v_ *β*_3_ (ITGB3), are promising targets due to their overexpression on both capillary endothelial cells (CECs) and ectopic endometrial stromal cells (EESCs) within EMS lesions.^[^ ^17^ ^]^ The cyclic pentapeptide cRGDyK (cRGD) demonstrates high affinity and specificity for ITGB3, facilitating receptor‐mediated endocytosis and transcytosis (RMT) for active lesion targeting.^[^ ^18^ ^]^ This targeting strategy aligns with emerging therapies for EMS, including photothermal therapy,^[^ ^19^ ^]^ ferroptosis induction,^[^ ^20^ ^]^ and immunotherapy.^[^ ^21^ ^]^ Among these, phototherapies, specifically photothermal therapy (PTT) and photodynamic therapy (PDT), offer tissue‐preserving ablation with established efficacy in antimicrobial and oncology applications.^[^ ^22^ ^]^ Their inherent safety, reproducibility, and cost‐effectiveness render them promising candidates for gynecological pathologies.^[^ ^23^ ^]^ PTT achieves localized ectopic lesion ablation via laser‐induced hyperthermia with minimal collateral damage, while PDT utilizes wavelength‐activated photosensitizers to generate cytotoxic reactive oxygen species (ROS; e.g., singlet oxygen).^[^ ^24^ ^]^ These ROS mediate targeted cellular destruction through oxidative biomolecular and microvascular disruption. Nevertheless, both modalities necessitate further mechanistic elucidation, efficacy optimization, and safety validation. The rapid systemic clearance of conventional probes further highlights the need for targeted theranostic agents capable of integrating diagnostic and therapeutic functions within a single platform.

**Figure 1:**
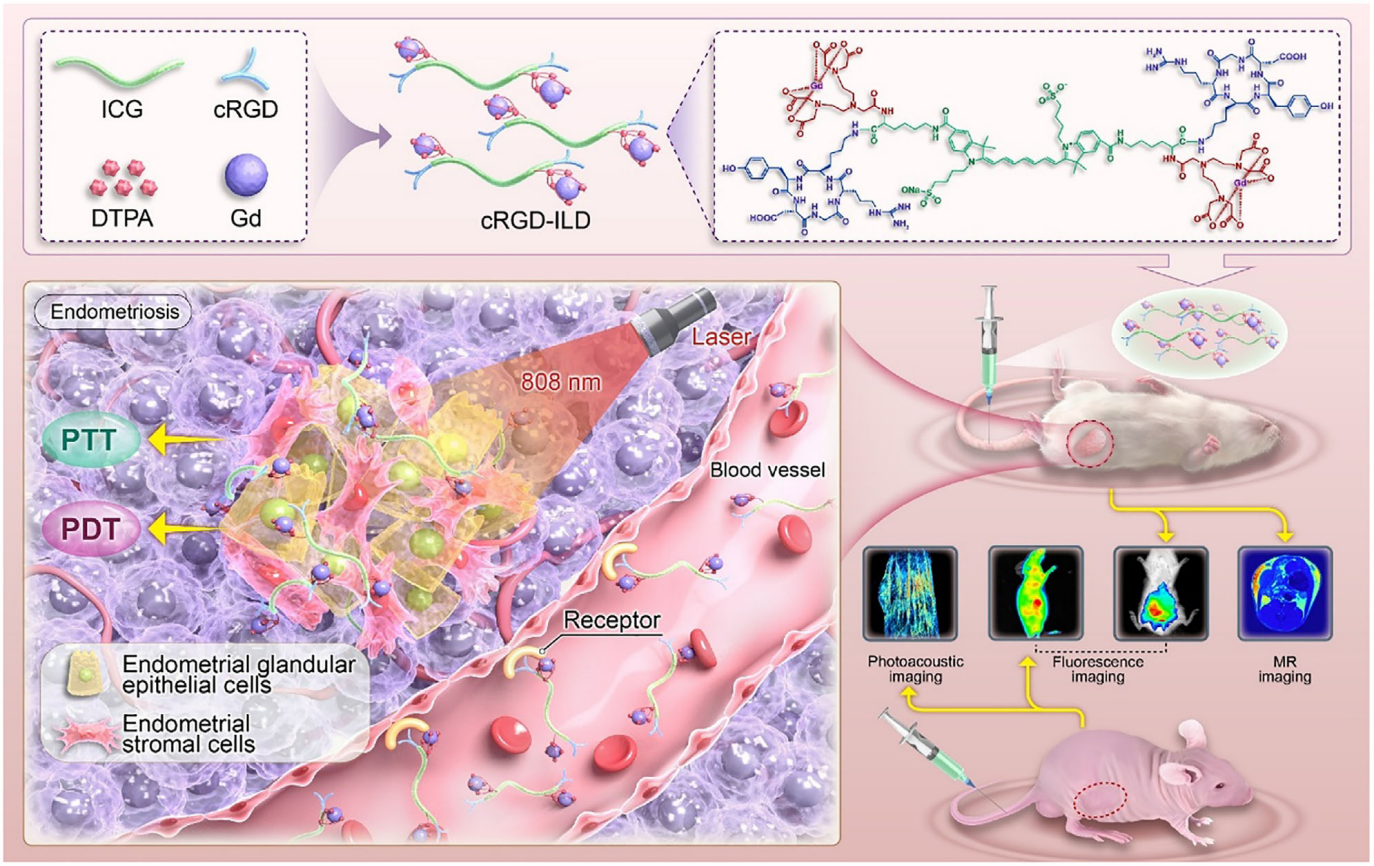
Schematic illustration of the cRGD‐ILD molecular probe for targeted endometriosis theranostics. The probe integrates a cyclic RGD peptide (integrin *α*_v_ *β*_3_‐targeting ligand), ICG (NIR dye enabling simultaneous photoacoustic/fluorescence imaging and phototherapy), and Gd‐DTPA (MRI contrast agent). Upon intravenous administration, integrin *α*_v_ *β*_3_ receptor‐mediated transcytosis drives selective accumulation in ectopic lesions. Under 808 nm laser irradiation, ICG mediates combined photothermal ablation and photodynamic ROS generation while facilitating photoacoustic and fluorescence imaging. Integrated with Gd‐enhanced MRI, this multimodal platform achieves precise lesion localization for image‐guided therapy.

To address the dual challenges of precise lesion identification and targeted therapy, we engineered a molecular probe specifically designed for multimodal EMS imaging and phototherapy. This probe integrates: i) the NIR photosensitizer indocyanine green (ICG), ii) gadolinium‐chelated diethylenetriaminepentaacetic acid (DTPA@Gd), and iii) the cRGD targeting peptide (**Figure** **1**). The probe achieves dual targeting of CECs and EESCs through cRGD‐ITGB3 binding. DTPA@Gd enables high‐resolution MRI for anatomical mapping, whereas ICG enables PAI‐based visualization of lesional vascularization and margins. This synergistic imaging platform provides unprecedented accuracy for lesion identification and guidance of subsequent PTT/PDT interventions. Under NIR irradiation, ICG concurrently generates localized hyperthermia (PTT) and cytotoxic ROS (PDT). Significantly, all components employ FDA‐approved materials (ICG, Gd‐chelated DTPA), ensuring established biosafety profiles and accelerating clinical translation potential. This integrated theranostic strategy represents a promising approach toward achieving more precise and effective management of EMS.

## 2 Results and Discussion

### 2.1 Characterization of cRGD‐ILD and ILD

**Figure 2:**
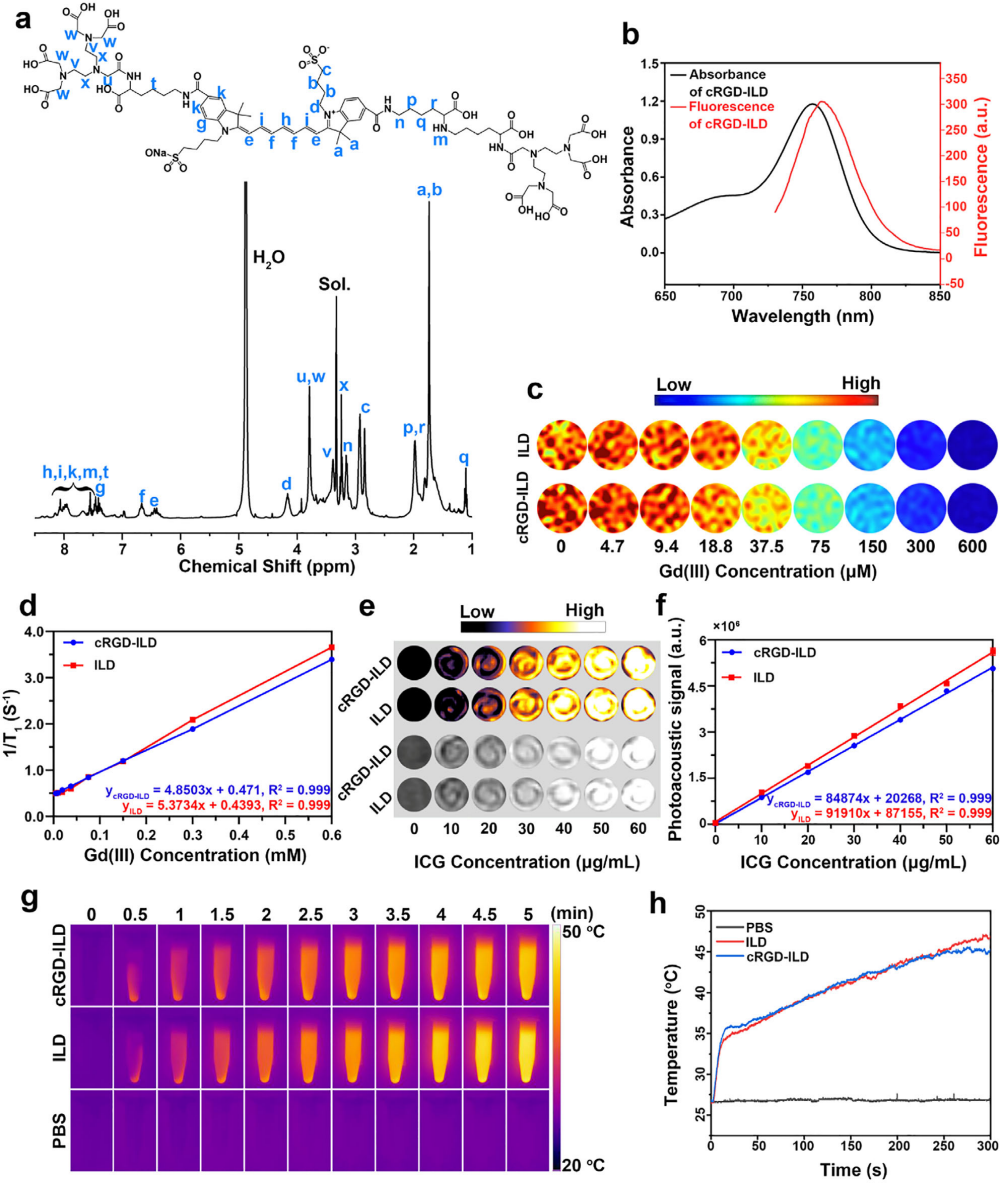
Characterization of cRGD‐ILD or ILD probe. a) The molecular structure of ICG‐lys‐DTPA verified by ^1^H NMR analysis. b) UV absorption spectrum and fluorescence emission spectrum (λ_ex_ = 755 nm) of cRGD‐ILD (*C*_ICG_ = 10 µg mL^−1^) in PBS. c) *T*_1_‐map images of ILD and cRGD‐ILD at various Gd concentrations (µM). d) *T*_1_ relaxation rates (s^−1^) as a function of Gd concentration (mM) for ILD and cRGD‐ILD, measured on a 3.0 T MRI scanner at room temperature. e) 3D‐view (up) and red slice view (down) of photoacoustic image of cRGD‐ILD and ILD solution at varying concentrations of ICG. f) The photoacoustic signal, measured as a function of ICG concentration (µg/mL), was evaluated for cRGD‐ILD and ILD using a TomoWave LOIS‐3D series PAI system. g) Infrared thermography of cRGD‐ILD, ILD, and PBS solutions under laser irradiation for different times (808 nm, 1.0 W cm^−2^, 5 min) and photothermal curves h) produced for the solutions at the corresponding time points. Data are presented as mean ± SD, n = 3 regions of interest (d, f).

The objective of this study was to develop innovative imaging and therapeutic agents capable of precisely identifying EMS lesions while reducing the adverse side effects associated with EMS treatment. To achieve this goal, we devised a strategy involving a molecular probe serving as a multimodal imaging platform to specifically target ectopic lesions and enhance the therapeutic efficacy of combined PTT/PDT against EMS. Consequently, the synthesis of the small molecular theranostic probes, namely cRGD‐ILD and ILD, was carried out according to the procedure outlined in Scheme S1 (Supporting Information). The synthesized products were subsequently verified by ^1^H nuclear magnetic resonance (^1^H NMR) and electrospray ionization mass spectrometry (ESI‐MS) (**Figure** **2**a; Figures S1–S8, Supporting Information). The characteristic peaks of ICG‐lys‐DTPA were found to align closely with the anticipated chemical shifts, as illustrated by the following ^1^H NMR (400 MHz, MeOD, 298 K) spectrum: δ (ppm) = 1.11 ppm (─NH─CH_2_CH_2_C ***H***_2_CH_2_─ of lys, q), 1.40‐1.81 ppm (─C(C ***H***_3_)_2_‐, a; ─CH_2_C ***H***_2_C ***H***_2_CH_2_SO_3_─, b), 1.87‐2.10 ppm (─NH─CH_2_C ***H***_2_CH_2_C ***H***_2_─ of lys, p, r), 2.93 ppm (CH_2_CH_2_CH_2_C ***H***_2_SO_3_─, c), 3.16 ppm (─NH─C ***H***_2_CH_2_CH_2_CH_2_─ of lys, n), 3.24 ppm (─N─(C ***H***_2_CH_2‐_N─(CH_2_COOH)_2_)_2_, x), 3.39 ppm (─N─(CH_2_C ***H***_2_─N─(CH_2_COOH)_2_)_2_, v), 3.60‐3.95 ppm (─N─(CH_2_CH_2_N(C ***H***_2_COOH)_2_)_2_, w; 3.94 ppm (─C ***H***_2_─N─(CH_2_CH_2_─N─(CH_2_COOH)_2_)_2_, u), 4.16 ppm (C ***H***_2_CH_2_CH_2_CH_2_SO_3_─, d), 6.44 ppm (─C═C ***H*** ─CH═CH─CH═CH─CH═C ***H*** ─C─, e), 6.66 ppm (─C═CH─CH═C ***H*** ─CH═C ***H*** ─CH═CH─C─, f), 7.37 ppm (─C ***H*** ─ of benzyl group, g), 7.40‐8.29 ppm (─C═CH─CH═CH─C ***H*** ═CH─CH═CH─C─, h; ─C═CH─C ***H*** ═CH─CH═CH─C ***H*** ═CH─C─, i; ─C ***H*** ─ of benzyl group, k; ─CO─N ***H*** ─(CH_2_)_4_─, m; ─N ***H*** ─DTPA, t).

The UV absorption and fluorescence emission spectra of the ICG and cRGD‐ILD probes were illustrated in Figure S9 (Supporting Information) and Figure 2b, respectively. Both ICG and cRGD‐ILD exhibited absorption peaks at 755 nm and fluorescence emission peaks at 764 nm. Given that MRI provides superior spatial resolution for evaluating EMS lesions, enabling comprehensive pelvic assessment with high soft‐tissue contrast essential for detecting malignant features and identifying extrapelvic involvement,^[^ ^25^ ^]^ we conducted in vitro acquisitions of *T*_1_‐weighted images (Figure S10, Supporting Information) and *T*_1_‐map images (Figure 2c) for ILD and cRGD‐ILD solutions at varying concentrations. Based on these images, *T*_1_ relaxation rates (s^−1^) were plotted against Gd concentration (mM) to ascertain *T*_1_ relaxivities (*r*_1_), yielding values of 4.85 mM^−1^·s^−1^ for cRGD‐ILD and 5.37 mM^−1^·s^−1^ for ILD (Figure 2d). Notably, these *r*_1_ values closely resembled that of the clinically approved Magnevist (5.35 mM^−1^·s^−1^),^[^ ^26^ ^]^ indicating the potential applicability of both cRGD‐ILD and ILD as effective *T*_1_‐weighted contrast agents. Since PAI demonstrated high accuracy in delineating lesion margins and characterizing vascular architecture in EMS‐affected tissues, providing enhanced visualization of histomorphological features,^[^ ^8b^ ^]^ we analyzed the PA properties of cRGD‐ILD using a TomoWave LOIS‐3D PAI system and LOIS View 3D software. Under 758 nm excitation, both cRGD‐ILD and ILD probes demonstrated concentration‐dependent enhancement of PA signal intensities correlating with ICG levels (Figure 2e,f), confirming their efficacy as PAI agents.

As a clinically approved phototheranostic agent, ICG serves as a cornerstone in PDT and PTT research.^[^ ^27^ ^]^ Whereafter, the ROS‐responsive indicator 3‐diphenylisobenzofuran (DPBF) was employed for the detection of ROS generation.^[^ ^28^ ^]^ As depicted in Figure S11a (Supporting Information), the normalized absorbance of DPBF at 410 nm decreased significantly with prolonged irradiation time. The efficient generation of ROS led to ≈58% and 56% reductions in DPBF absorbance within 10 min for cRGD‐ILD and ILD solutions, respectively, under 808 nm laser irradiation (Figure S11b,c, Supporting Information). The photothermal effects of the probes were quantified in Figure 2g,h. After 5 min of irradiation with an 808 nm laser, both cRGD‐ILD and ILD solutions underwent a marked temperature elevation of ≈20 °C, surpassing the 42 °C threshold for cellular cytotoxicity.^[^ ^29^ ^]^ In stark contrast, the PBS control solution exhibited a negligible thermal response, with a mere 0.2 °C increase. Photothermal conversion efficiency (PCE) and stability were further evaluated to assess the probe's therapeutic potential. The cRGD‐ILD solution reached a maximum temperature increase of ≈45 °C (Figure S12a, Supporting Information), with a calculated PCE of 29.81% (Figure S12b and calculation method in Figure S12c, Supporting Information). This value significantly exceeds that of free ICG (15.8%),^[^ ^30^ ^]^ suggesting enhanced therapeutic efficacy. Meanwhile, both cRGD‐ILD and ILD solutions exhibited a concentration‐dependent temperature elevation under laser irradiation (Figure S13a,b, Supporting Information). This multimodal performance aligns with emerging theranostic trends where integrated imaging/therapy platforms show superior clinical utility over single‐modality agents.^[^ ^31^ ^]^

### 2.2 Quantitative Analysis of Probe Internalization

**Figure 3:**
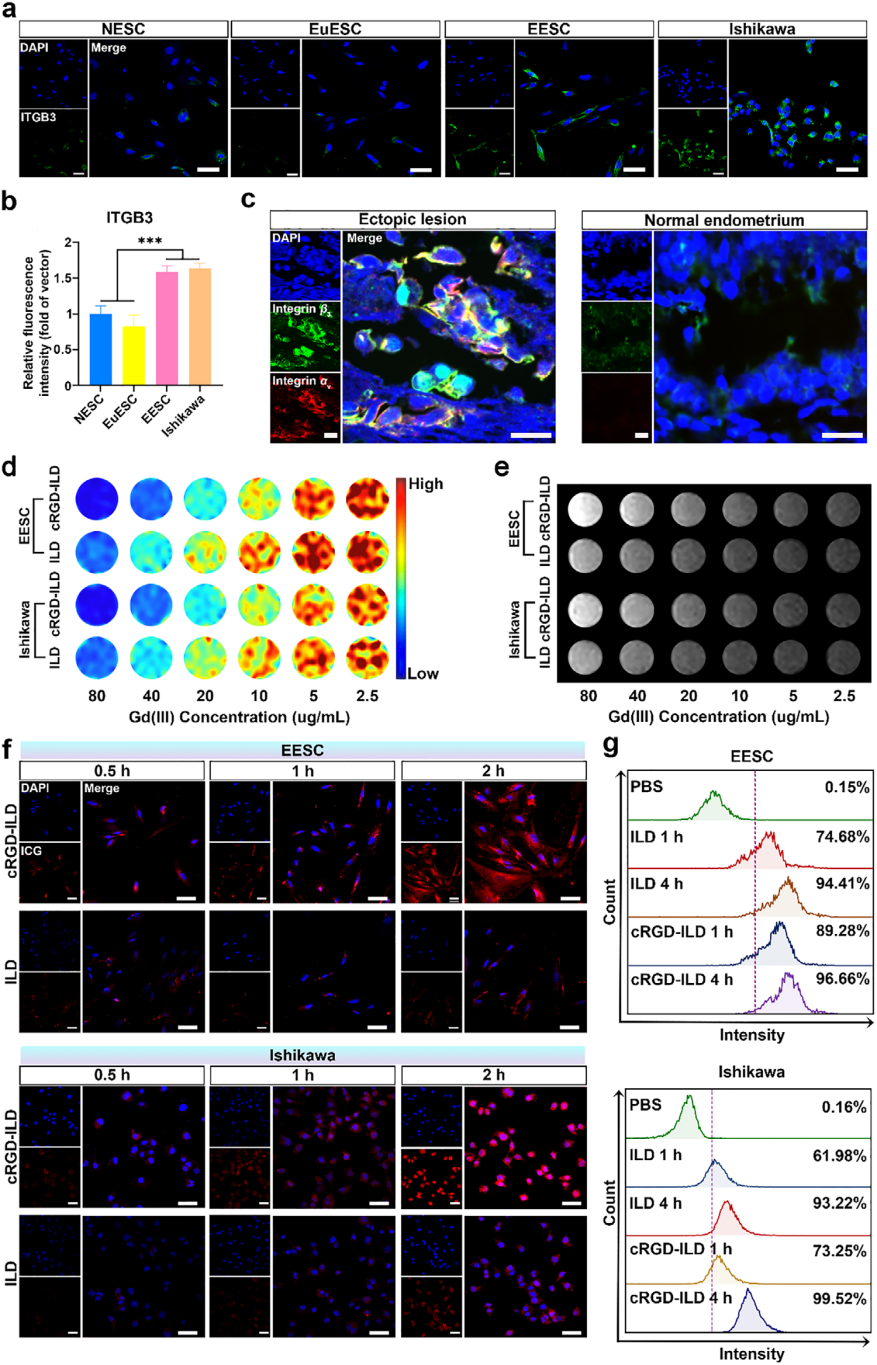
Expression levels of integrin *α*_V_ and *β*_3_ in EMS and cellular uptake of molecular probes. a) Typical immunofluorescence images showing ITGB3 expression (green fluorescence) in normal endometrial stromal cells (NESC), eutopic endometrial stromal cells (EuESC), ectopic endometrial stromal cells (EESC), and Ishikawa cells. Scale bar: 50 µm. b) Quantification of ITGB3 expression levels from (a). Data are presented as mean ± SD, n = 3 per group. Statistical significance was determined by one‐way ANOVA with LSD post‐hoc test; ^***^ *p* < 0.001. c) Typical immunofluorescence images of integrin *α*_V_ and *β*_3_ expression in ectopic lesions from EMS patients and normal endometrium from non‐EMS patients. Scale bar: 20 µm. d) *T*_1_‐map and e) *T*_1_‐weighted MR images of Ishikawa cells and EESCs after 2 h incubation with ILD and cRGD‐ILD at indicated Gd concentrations (µg/mL). f) Fluorescence images of EESCs and Ishikawa cells incubated with cRGD‐ILD and ILD at different time points under CLSM observation. Scale bar: 50 µm. g) Quantification of internalized probes in EESCs and Ishikawa cells by flow cytometry.

To investigate molecular probe internalization, we employed an in vitro model utilizing EESCs and Ishikawa cells. As a well‐differentiated endometrial adenocarcinoma cell line, Ishikawa cells serve as an established in vitro model for studying endometrial epithelial physiology, notably due to their characteristic high expression of ITGB3^[^ ^32^ ^]^ and frequent use in EMS research.^[^ ^33^ ^]^ Our analysis revealed ubiquitous vimentin expression across all primary endometrial stromal cells (ESCs), while cytokeratin marker expression was negligible (Figure S14, Supporting Information). In contrast, both EESCs and Ishikawa cells exhibited significantly higher ITGB3 expression levels compared to normal endometrial stromal cells (NESCs) and eutopic endometrial stromal cells (EuESCs) (**Figure** **3**a,b). Complementary immunofluorescence staining further confirmed that integrin *α*_V_ and *β*_3_ levels were markedly elevated in ovarian endometrioma tissues from EMS patients relative to normal endometrial tissues from non‐EMS controls (Figure 3c; Figure S15a,b, Supporting Information). Consistent with these findings, immunohistochemical staining analyses demonstrated a significant upregulation of both integrin *α*_V_ and *β*_3_ subunits in ectopic lesions of EMS rats compared to normal endometrial tissue (Figure S16a–c, Supporting Information).

The internalization of probes into cells was confirmed by MRI. As shown in Figure 3d,e, the *T*_1_‐weighted MRI signal intensity, which corresponds to the brightness in *T*_1_‐weighted images, increased in cells treated with either cRGD‐ILD or ILD across Gd (III) concentrations ranging from 2.5 to 80 µg mL^−1^. Notably, cells incubated with cRGD‐ILD exhibited higher *T*_1_‐weighted signal intensities coupled with shorter *T*_1_ longitudinal relaxation times compared to those incubated with ILD, confirming the enhanced targeting efficacy achieved through cRGD‐mediated active delivery mechanisms. Internalization was further analyzed by confocal laser scanning microscopy (CLSM) and flow cytometry. After 0.5 h incubation, EESCs and Ishikawa cells treated with cRGD‐ILD displayed significantly stronger intracellular red fluorescence (ICG) than ILD‐treated cells (Figure 3f). Flow cytometry corroborated these findings (Figure 3g), collectively demonstrating cRGD‐ILD's superior cellular uptake mediated by cRGD targeting.

### 2.3 In Vitro ROS Detection and Anti‐EMS Efficacy

**Figure 4:**
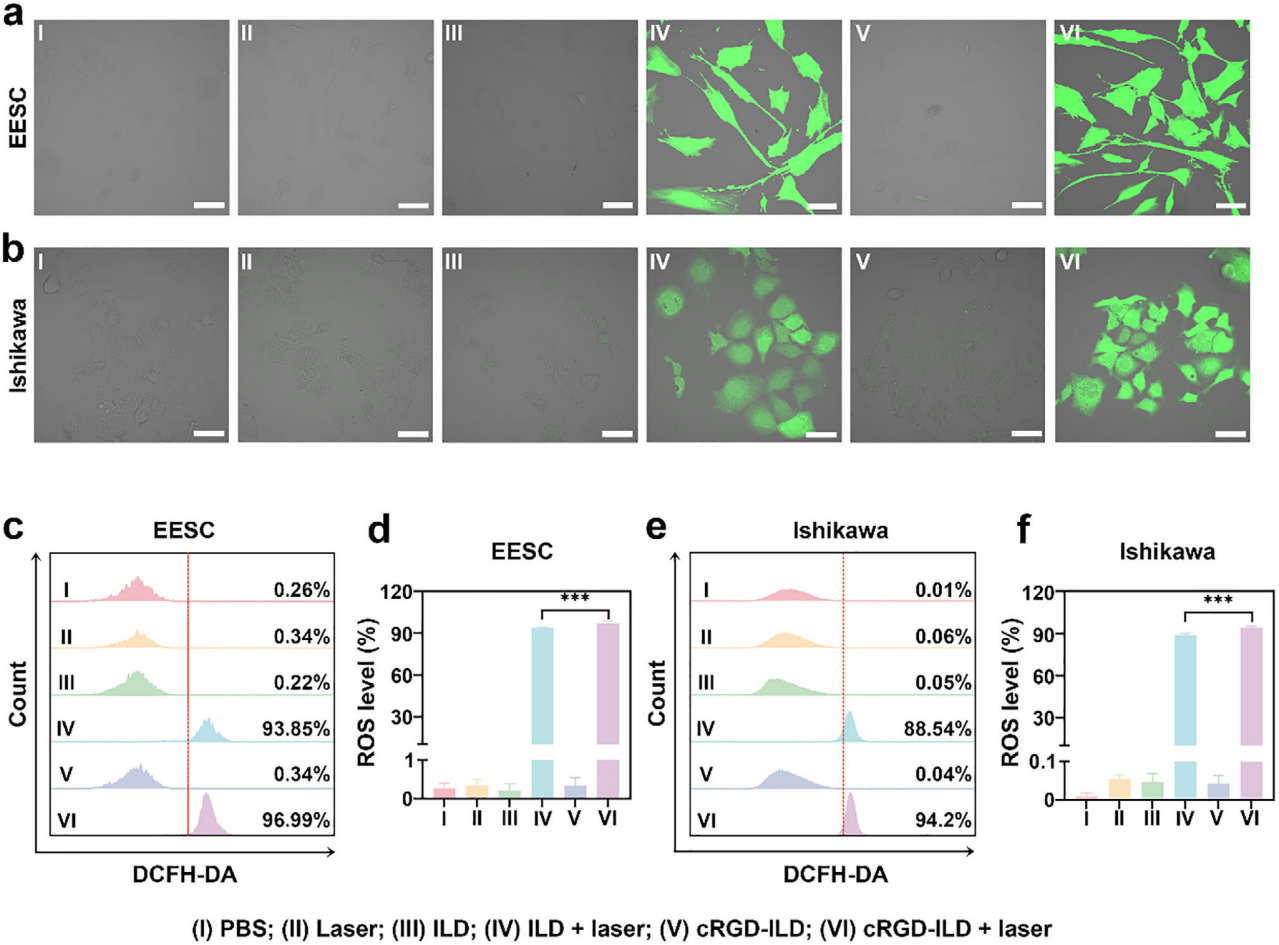
In vitro PDT effects of cRGD‐ILD and ILD probes. a,b) CLSM images showing intracellular ROS generation in EESCs (a) and Ishikawa cells (b) after treatment. Scale bar: 100 µm. c) Flow cytometry analysis of intracellular ROS levels in EESCs. d) Quantitative analysis of ROS levels from flow cytometry in EESCs across treatment groups. e) Flow cytometry analysis of intracellular ROS levels in Ishikawa cells. f) Quantitative analysis of ROS levels from flow cytometry in Ishikawa cells across treatment groups. Data are presented as mean ± SD, n = 4 independent samples/group. Statistical significance was calculated by one‐way analysis of variance and LSD post‐hoc test; ^***^ *p* < 0.001.

The intracellular ROS generation in EESCs and Ishikawa cells was evaluated using the 2′,7′‐dichlorodihydrofluorescein diacetate (DCFH‐DA) assay, as shown in **Figure** **4**a,b. Synergistic treatment combining molecular probe incubation with laser irradiation resulted in a robust ROS signal amplification within the cells, whereas independent applications of either laser exposure or molecular probe administration alone produced minimal, statistically insignificant elevations in intracellular ROS levels. Furthermore, quantitative analysis via flow cytometry revealed that EESCs treated with PBS, ILD + laser, and cRGD‐ILD + laser exhibited normalized fluorescence intensities of 0.26%, 93.85%, and 96.99%, respectively (Figure 4c,d). Analogous trends were observed in Ishikawa cells, with corresponding values of 0.01%, 88.54%, and 94.2%, respectively (Figure 4e,f). These results substantiate that enhanced cellular internalization of cRGD‐ILD facilitates superior ROS generation efficiency under NIR irradiation, which is crucial for maximizing therapeutic efficacy in EMS management.

**Figure 5:**
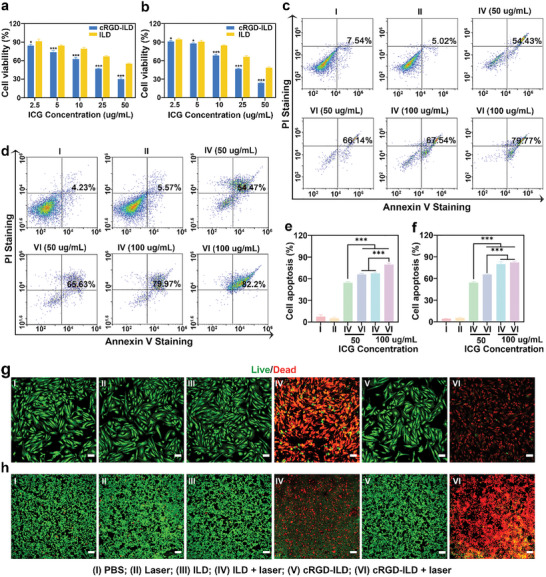
In vitro anti‐EMS efficacy of cRGD‐ILD and ILD probes. a,b) Viability of EESCs (a) and Ishikawa cells (b) treated with cRGD‐ILD or ILD followed by 808 nm laser irradiation (1.0 W cm^−^^2^, 5 min), assessed by CCK‐8 assay. c,d) Flow cytometry analysis of apoptosis in EESCs (c) and Ishikawa cells (d) under identical treatment conditions. e,f) Quantitative analysis of apoptotic rates in EESCs (e) and Ishikawa cells (f) from (c, d) across treatment groups. g,h) Live/dead staining with Calcein‐AM (green, viable cells) and propidium iodide (PI; red, dead cells) of EESCs (g) and Ishikawa cells (h) after probe incubation and laser irradiation. Scale bar: 100 µm. Data are presented as mean ± SD, n = 4 independent cell samples per group. Statistical significance was calculated by two‐tailed unpaired Student's *t*‐test (a, b) or one‐way analysis of variance and LSD post‐hoc test (e, f); ^*^ *p* < 0.05, ^***^ *p* < 0.001.

The cytotoxic effects of the probes were initially assessed using a Cell Counting Kit‐8 (CCK‐8) assay in EESCs and Ishikawa cells. As depicted in Figure S17 (Supporting Information), both cRGD‐ILD and ILD exhibited minimal cytotoxicity in EESCs and Ishikawa cells. After a 24 h incubation period without laser irradiation, the cell viability remained above 90% even at an ICG concentration up to 300 µg mL^−1^. Conversely, upon laser irradiation (808 nm, 1.0 W cm^−^^2^, 5 min), the two probes exhibited a significant and concentration‐dependent enhancement of cytotoxic potency in both EESCs and Ishikawa cells (**Figure** **5**a,b). Specifically, cRGD‐ILD exhibited significantly higher cytotoxicity than ILD, with cell viability of 30.27 ± 2.02% for cRGD‐ILD versus 55.35 ± 1.1% for ILD in EESCs, and 23.8 ± 1.36% for cRGD‐ILD versus 48.41 ± 1.74% for ILD in Ishikawa cells, respectively. This disparity may be attributed to the enhanced cellular accumulation of cRGD‐ILD, as previously demonstrated. Additionally, cell apoptosis was evaluated using the Annexin V‐fluorescein isothiocyanate (FITC)/propidium iodide (PI) Apoptosis Detection Kit, and the findings corroborated the cell viability results (Figure 5c–f).

Next, an additional calcein‐AM/PI co‐staining assay was conducted to evaluate the therapeutic efficacy against EMS of the probes. In this assay, red fluorescence signified dead cells stained with PI, whereas green fluorescence represented living cells stained with calcein‐AM. As depicted in Figure 5g,h, the cells treated with cRGD‐ILD and ILD in the absence of 808 nm laser irradiation exhibited minimal red fluorescence, indicating negligible cell death. Conversely, pronounced red fluorescence was observed in EESCs and Ishikawa cells treated with cRGD‐ILD and ILD under 808 nm laser irradiation. Notably, incubation with cRGD‐ILD combined with 808 nm laser irradiation yielded the strongest red fluorescence, indicating the effective anti‐EMS impact of cRGD‐ILD attributable to its enhanced targeting of EMS cells.

The combined PTT/PDT strategy effectively mitigates the inherent limitations of each individual modality. While PDT alone requires prolonged illumination and is intrinsically limited by hypoxia, PTT alone is hampered by inadequate heat confinement and thermal damage to surrounding tissues due to the temperature gradients.^[^ ^34^ ^]^ Our dual‐modality strategy achieves rapid thermal ablation while simultaneously inducing ROS‐mediated cytotoxicity through synergistic interactions.

### 2.4 In Vivo Biodistribution of Molecular Probes

**Figure 6:**
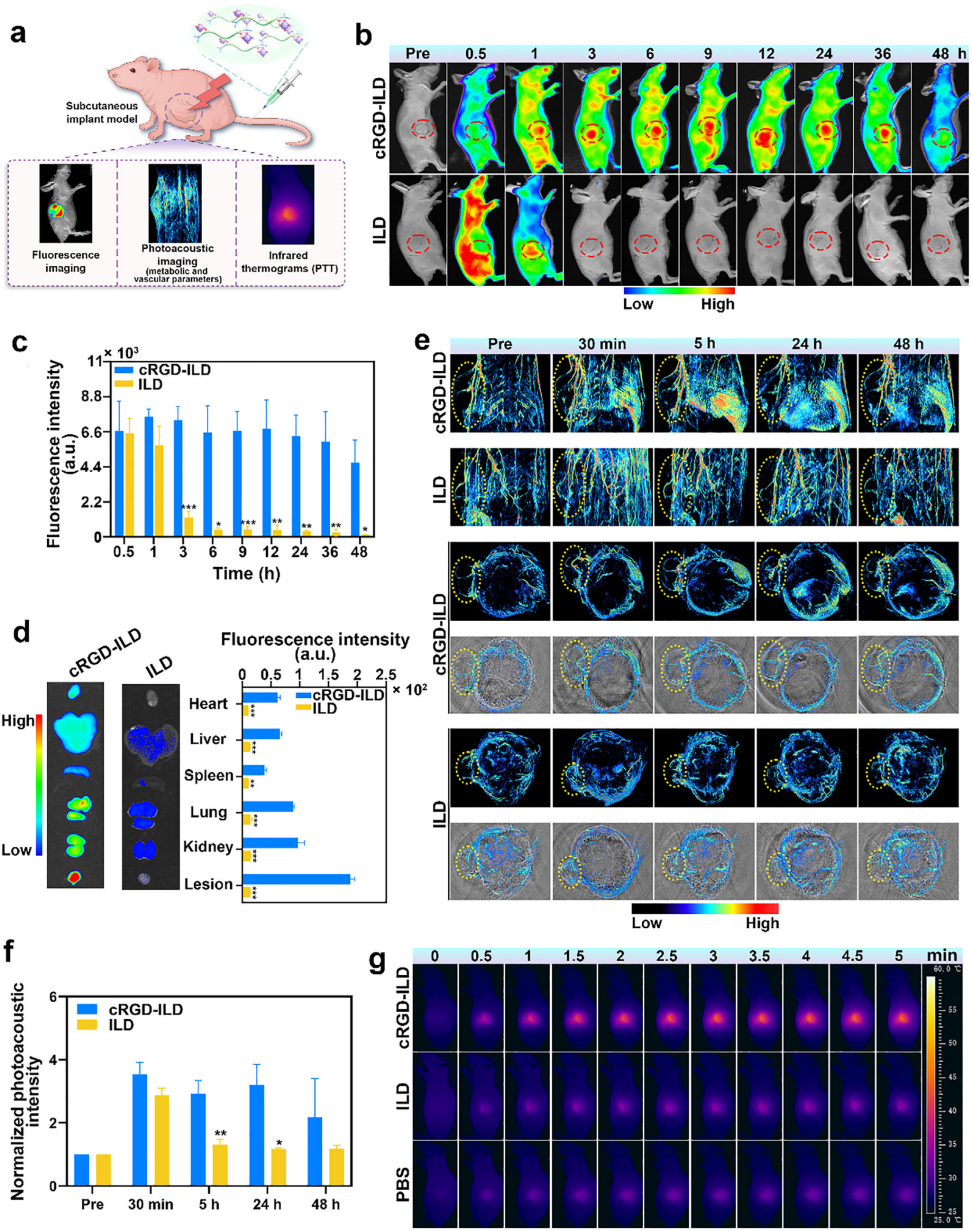
In vivo multimodal imaging and photothermal performance of molecular probes in EMS model mice. a) Schematic of fluorescence, photoacoustic (PA), and photothermal imaging in a subcutaneously transplanted nude mouse model of EMS following intravenous administration of molecular probes via the tail vein. b) In vivo fluorescence images at various time points post‐injection of cRGD‐ILD or ILD. Red circles denote lesion regions. c) Time‐dependent mean fluorescence intensity at EMS lesion sites. d) Ex vivo fluorescence images and quantitative analysis (mean intensity) of lesions and major organs harvested 48 h post‐injection. e) Axial (up) and coronal (down) PA images post‐injection. Axial PA images following injection of cRGD‐ILD (Movie S1, Supporting Information) and ILD at 24 h (Movie S2, Supporting Information). Yellow circles delineate lesion locations. f) Normalized PA signal intensities in lesions over time post‐injection. g) Photothermal images of EMS lesions irradiated with an 808 nm laser (1.0 W cm^−^^2^, 5 min) at different time points post‐injection of cRGD‐ILD, ILD, or PBS. Data are presented as mean ± SD, n = 3 mice per group. Statistical significance was calculated by a two‐tailed unpaired Student's *t*‐test; ^*^ *p* < 0.05, ^**^ *p* < 0.01, ^***^ *p* < 0.001 (compared with cRGD‐ILD group).

In vivo fluorescence imaging was carried out to investigate the accumulation capacity of cRGD‐ILD at ectopic lesion sites in EMS nude mice. After successfully establishing a subcutaneous xenograft model of EMS mice for 21 days, the cRGD‐ILD and ILD molecular probes were administered through the tail vein. Due to interference from rat hair with photoacoustic (PA) signal acquisition and monitoring of local temperature elevation at the lesion site, fluorescence, PA, and photothermal imaging were performed using a subcutaneously transplanted nude mouse model (**Figure** **6**a). PA imaging provides valuable information on metabolic and vascular parameters, while photothermal imaging facilitates the assessment of PTT efficacy. As shown in Figure 6b,c, cRGD‐ILD demonstrated significantly enhanced accumulation efficiency at ectopic lesion sites coupled with a marked prolongation of drug circulation time in vivo. In stark contrast, ILD‐treated mice exhibited negligible ICG accumulation at the target lesion sites, with the fluorescent signal rapidly diminishing within the body. Moreover, the fluorescence imaging results of isolated lesions were consistent with these observations (Figure 6d), indicating that cRGD‐ILD can be taken up by the tissue of ectopic lesions in EMS. These findings clearly demonstrate the unique capability of cRGD‐ILD to sequentially target ectopic lesions, enabling the probe to accumulate preferentially in EMS tissue.

The in vivo PAI capability of the probe was evaluated using a TomoWave LOIS‐3D series PAI system. The molecular probe was administered intravenously via the tail vein in EMS‐bearing mice. As illustrated in Figure 6e, pre‐administration imaging revealed indistinct lesion boundaries in both cRGD‐ILD and ILD groups. Subsequent to intravenous injection, a marked augmentation of PA signals was observed in lesional regions of both groups at 0.5 h post‐administration (Figure 6f). Divergent pharmacokinetic profiles emerged over temporal progression: The cRGD‐ILD cohort sustained elevated PA signal intensities at 5 and 24 h post‐injection (Movie S1, Supporting Information), while the ILD group demonstrated rapid signal attenuation with significantly compromised imaging resolution by 5 h, attributable to accelerated metabolic clearance. Longitudinal monitoring revealed that lesion‐to‐margin signal intensities in the ILD group regressed to baseline levels at 24 h (Movie S2, Supporting Information) and 48 h timepoints. Notably, PA signal diminution in the cRGD‐ILD treated group manifested exclusively at 48 h post‐intervention. Collectively, these findings underscore the targeted lesion‐specific accumulation of cRGD‐ILD, which serves to amplify PAI detection sensitivity and facilitate clearer imaging of both lesion boundaries and their associated vascular networks.

Given PTT's efficacy as a minimally invasive, selective cellular ablation method using heat from light‐activated, non‐toxic agents,^[^ ^35^ ^]^ combined with fluorescence‐guided surgery for precise lesion targeting, which shows potential to reduce EMS post‐operative recurrence,^[^ ^35b^ ^]^ we used infrared thermography to monitor local lesion warming in cRGD‐ILD and ILD‐injected mice under 808 nm laser exposure. As depicted in Figure 6g and Figure S18 (Supporting Information), cRGD‐ILD exhibited selective accumulation at the ectopic lesion site of EMS, achieving a localized temperature up to 45.6 °C within 5 min of laser irradiation and enabling efficient thermal ablation. Conversely, ILD showed reduced lesion targeting, achieving a maximum temperature of only 34.5 °C. Spatially confining laser irradiation to the lesion site generates higher target temperatures while keeping surrounding tissues significantly cooler, thereby preventing off‐target damage. In contrast, the control group administered PBS exhibited a modest temperature elevation (32.5 °C), while NIR laser irradiation (808 nm, 1 W cm^−^^2^) following PBS administration produced a minimal 3 °C temperature increase in endometriotic grafts. This negligible thermal response confirms the inherent safety of the adopted photothermal parameters, ensuring specific heating confined to targeted tissues throughout the therapeutic window. These findings demonstrate that systemic administration of cRGD‐ILD allows for effective accumulation in EMS lesions, provides high EMS lesion‐to‐normal tissue contrast through multimodal imaging, and generates high temperatures in EMS lesions upon targeted light exposure.

**Figure 7:**
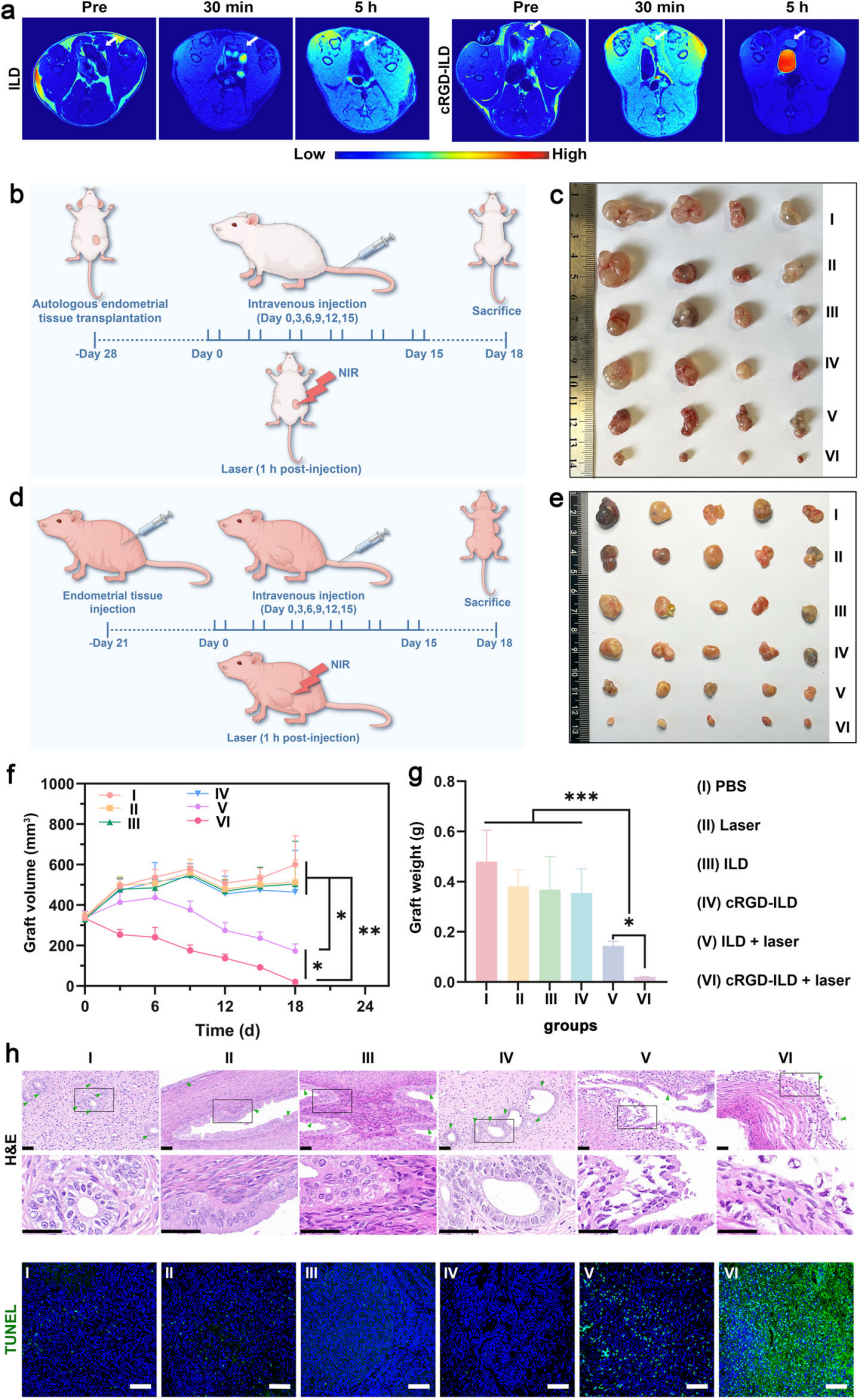
In vivo MR images and therapeutic efficacy of molecular probes in both autografted EMS rats and xenografted nude mice. a) Transverse *T*_1_‐weighted MR images of EMS lesions post‐injection. White arrows indicate lesion locations. b) Schematic of PDT/PTT therapeutic protocol for autotransplanted EMS rats. c) Photographs of endometriotic lesions excised from rats 18 days post‐treatment. d) Schematic of the experimental design for PDT/PTT therapies in the xenograft EMS mouse model. e) Photographs of endometriotic lesions excised from nude mice 18 days post‐treatment. f) Mean lesion volumes and g) mean lesion weights in EMS‐bearing mice across treatment groups. h) Histological analysis (scale bar: 40 µm) and TUNEL assay (scale bar: 100 µm) of ectopic lesion tissues. Endometrioid glands are indicated by arrowheads. Data are presented as mean ± SD, n = 4 rats (or 5 mice) per group. Statistical significance was calculated by a two‐tailed unpaired Student's *t*‐test (f) or one‐way analysis of variance and LSD post‐hoc test (g); ^*^ *p* < 0.05, ^**^ *p* < 0.01, ^***^ *p* < 0.001.

To further validate the imaging capability of molecular probes across diverse animal models, we established an additional autologous EMS rat model. At 28 days post‐modeling, we assessed the biodistribution of molecular probes at lesion sites and their efficacy in enhancing MRI through in vivo fluorescence imaging and MRI scans, respectively. Consistent with prior observations, in vivo fluorescence scanning revealed that cRGD‐ILD achieved higher targeting efficiency to ectopic lesion sites in autologous EMS rats (Figure S19a–d, Supporting Information), confirming the species‐independent universality of its EMS‐targeting potential. On the other hand, MRI detected no significant increase in *T*_1_‐weighted signal intensity at the lesion site of ILD‐treated rats (**Figure** **7**a). In contrast, cRGD‐ILD‐administered rats exhibited a notable enhancement in *T*_1_‐weighted signal intensity at both 30 min and 5 h post‐injection, as demonstrated in cross‐sectional views. These results establish cRGD‐ILD as a potent MRI contrast agent, highlighting its potential to improve clinical EMS detection through enhanced imaging contrast.

The nude mouse xenograft model, despite lacking an intact immune system, enables clear visualization of lesion‐specific targeting.^[^ ^19^ ^]^ The autologous rat model better recapitulates human EMS pathophysiology via transplantation of autologously derived endometrial tissue^[^ ^36^ ^]^ and demonstrates comparable biodistribution patterns. Collectively, these models validate the translational potential, as lesion targeting was maintained across species (mouse to rat) and implantation methods (xenograft vs autograft).

### 2.5 In Vivo Anti‐EMS Effect

The successful delivery of molecular probes to lesion sites prompted further evaluation of their therapeutic potential for EMS. PTT and PDT have garnered significant attention in the realm of accurate and safe management of gynecological disorders, owing to their noninvasive nature and high selectivity.^[^ ^23^ ^]^ Thus, the effectiveness of in vivo phototherapy was evaluated in rats and nude mice harboring ectopic endometrial tissues using an 808 nm NIR beam optimized for effective abdominal tissue penetration. Rat models bearing ectopic endometrial implants were randomly assigned to six groups (n = 4) for the following treatments of PBS, NIR laser irradiation (laser), ILD, cRGD‐ILD, ILD plus NIR laser irradiation (ILD + laser), and cRGD‐ILD plus NIR laser irradiation (cRGD‐ILD + laser). These therapeutic agents were intravenously administered to the rats 28 days after autologous endometrial tissue transplantation, with each administration occurring once every 3 days for a total of six times. For the groups receiving NIR laser irradiation, the procedure was performed 1 h post‐administration at a power of 1.0 W cm^−^^2^ for 10 min (Figure 7b). In the autologous transplantation model of EMS in rats, the groups treated with probes in conjunction with NIR laser irradiation, specifically the cRGD‐ILD + laser group and the ILD + laser group, demonstrated superior efficacy in inhibiting lesion growth compared to the other groups (Figure 7c; Figure S20a,b, Supporting Information). Notably, the therapeutic effect of the cRGD‐ILD + laser combination was even more pronounced than that of the ILD + laser treatment alone. In contrast, the lesion growth rate was significantly faster in the remaining four groups, indicating their insignificant anti‐EMS effects.

Drawing parallels with the rat model, we developed a xenograft nude mouse model for EMS and applied identical therapeutic protocols (Figure 7d). Notably, the xenograft EMS nude mouse cohort demonstrated remarkably superior treatment responses (Figure 7e–g). Specifically, the phototherapy regimen combining cRGD‐ILD with laser treatment outperformed the ILD + laser combination in terms of therapeutic efficacy. The application of NIR light (>700 nm) presents notable advantages over visible light (400–700 nm), such as improved tissue compatibility, a higher maximum permissible exposure limit, and greater penetration depth, which is facilitated by reduced absorption by hemoglobin and water.^[^ ^37^ ^]^ Collectively, these attributes facilitate substantial therapeutic benefits even in the demanding setting of abdominal autologous transplant models, thereby highlighting the promising clinical potential of NIR‐mediated phototherapy for treating deep‐seated lesions.

Histopathological examination of ectopic lesions via hematoxylin and eosin (H&E) staining (Figure 7h) revealed pronounced graft growth suppression in mice receiving ILD + laser and cRGD‐ILD + laser treatments compared to controls, with the cRGD‐ILD + laser cohort exhibiting the most significant alterations: a reduction in cellular density, disorganized glandular epithelium accompanied by a decrease in gland count, and marked stromal thinning. Consistent with these findings, TUNEL assays confirmed peak apoptotic activity in cRGD‐ILD + laser‐treated lesions, collectively validating cRGD‐ILD as a potent theranostic platform for MRI/PAI/fluorescence‐guided phototherapy against EMS. The integrin *α*_V_ *β*_3_ targeting strategy employed here could be adapted for other diseases characterized by integrin overexpression. For instance, in rheumatoid arthritis, osteoclasts and synovial inflammatory macrophages also show elevated *α*_V_ *β*_3_ expression,^[^ ^38^ ^]^ suggesting potential applications beyond EMS. Furthermore, the modular design allows substitution of the targeting ligand (cRGD) with other disease‐specific markers, enabling platform adaptation for diverse pathologies.

Throughout the treatment period, body weights remained comparable across all experimental groups in both rats and mice (Figures S21 and S22, Supporting Information), with no statistically significant intergroup differences. To evaluate off‐target effects and clearance kinetics, healthy BALB/c nude mice were administered cRGD‐ILD intravenously via the tail vein. Serial in vivo fluorescence imaging at various time points showed no specific tissue distribution. The fluorescence signal declined to ≈14.75% of the 24 h level within 7 days, demonstrating significant metabolic clearance of this small‐molecule drug (Figure S23a,b, Supporting Information). Healthy nude mice were sacrificed 10 days post‐injection. Ex vivo fluorescence imaging of major organs (heart, liver, spleen, lungs, and kidneys) revealed minimal residual fluorescence (Figure S23c,d, Supporting Information). Hematological and biochemical analyses further demonstrated intact physiological parameters, as evidenced by unaltered blood cell counts and normal hepatic/renal function markers in all cohorts (Figures S24 and S25, Supporting Information). Histopathological examination of major organs (heart, liver, spleen, lungs, kidneys, and uterus) revealed no detectable alterations or pathological abnormalities in any treatment group (Figure S26, Supporting Information). These integrated safety evaluations collectively demonstrate the favorable biosafety profiles of the administered probes.

Several pathways emerge for clinical adoption: serving as a preoperative imaging adjunct to conventional ultrasound/MRI to improve surgical planning through precise lesion delineation; functioning as an intraoperative guidance tool for fluorescence‐guided resection to potentially reduce recurrence rates;^[^ ^39^ ^]^ and offering a standalone therapeutic alternative to surgery or traditional hormone therapy. The observed safety profile, supported by normal organ histopathology, provides a foundation for advancing toward good manufacturing practice production and facilitates required toxicology studies for investigational new drug applications.

## 3 Conclusion

A theranostic probe (cRGD‐ILD) was developed through integrating a *T*_1_‐weighted MRI contrast agent, ICG, and a cRGD peptide to enable simultaneous integrin receptor‐mediated targeting of both CECs and endometriotic lesions. This water‐soluble molecular probe exhibits high transluminal permeability via ITGB3‐mediated RMT, which facilitates a multimodal MR/PA/fluorescence imaging of EMS lesions. In preclinical EMS models (xenograft nude mice and autologous rats), cRGD‐ILD demonstrated species‐independent lesion accumulation and superior uptake by EESCs for highly efficient PTT/PDT under deep‐tissue‐penetrating NIR irradiation. The modular probe, combining FDA‐approved components with clinically translatable imaging capabilities, offers transformative potential for image‐guided EMS management via enabling real‐time therapeutic monitoring and optimized intervention strategies.

## 4 Experimental Section

### Reagents

The cRGD peptide (cyclic RGDyK, sequence Arg‐Gly‐Asp‐D‐Phe‐Lys), exhibiting a remarkable purity of 95.71%, was sourced from Wuhan Haode Biotechnology Co., Ltd. (Wuhan, China). Other chemicals, including 4‐hydrazinobenzoic acid, 3‐methyl‐2‐butanone, sodium acetate, glacial acetic acid, 1,4‐butane sultone, 1,2‐dichlorobenzene, and diethylenetriaminepentaacetic dianhydride (DTPAA), were procured from Aladdin Industrial Corporation (Shanghai, China) and were used without further modification. Glutacondianil hydrochloride, nalpha‐Fmoc‐L‐lysine hydrochloride, and GdCl_3_•6H_2_O were purchased from J&K Scientific Ltd. (Beijing, China). Additionally, N‐hydroxysuccinimide (NHS), 1‐(3‐dimethylaminopropyl)‐3‐ethylcarbodiimide hydrochloride (EDC•HCl), 4‐dimethylaminopyridine (DMAP), DPBF, and anhydrous dimethylsulfoxide (DMSO) were obtained from Sigma‐Aldrich (St. Louis, MO, USA). A dialysis bag was purchased from Green Bird Technology Development Co., Ltd. (Shanghai, China). Lastly, chemicals such as diethyl ether, methanol, acetone, acetic anhydride, 1‐propanol, piperidine, and NaHCO_3_ solid were acquired from Chemical Reagent Factory (Guangzhou, China).

Dulbecco's Modified Eagle Medium (DMEM) culture medium, DMEM/Nutrient Mixture F‐12 (DMEM/F12) culture medium, phosphate‐buffered saline (PBS), 0.25% trypsin‐EDTA, and fetal bovine serum (FBS) were procured from ThermoFisher Scientific (Gibco, USA). Type I collagenase was procured from Solarbio Technology Co., Ltd. (Beijing, China). The 1% penicillin/streptomycin solution was obtained from Guangzhou Danmai Biotechnology Co., Ltd. (Gibco, USA). Bovine serum albumin (BSA) was procured from Guangzhou Saiguo Biotech Co., Ltd. (Biofroxx, Germany). Additionally, 4′,6′‐diamidino‐2‐phenylindole (DAPI) and CCK‐8 were purchased from Guangzhou Chenxing Biotechnology Development Co., Ltd. (Biosharp, China). The Live & Dead Viability/Cytotoxicity Assay Kit was sourced from KeyGEN BioTECH Co., Ltd. (Nanjing, China). ROS assay reagent, specifically DCFH‐DA, was acquired from Sigma–Aldrich (St. Louis, MO, USA). β‐estradiol 17‐pentanoate was procured from McLean Biochemical Science and Technology Co., Ltd. (Shanghai, China). All remaining reagents employed in this study were commercially sourced and of analytical purity grade or superior.

### Preparation of 2,3,3‐Trimethyl‐3H‐indole‐5‐carboxylic Acid (TICA)

The water‐soluble molecular probes were synthesized in accordance with the methodology delineated in Scheme S1 (Supporting Information). ICG, featuring double carboxyl groups, was initially synthesized with minor modifications based on a previous report.^[^ ^40^ ^]^ Initially, the synthesis of TICA commenced with polymerization by mixing sodium acetate (4.97 g, 60 mmol), 3‐methyl‐2‐butanone (3.65 g, 42 mmol), and 4‐hydrazinobenzoic acid (4.65 g, 30 mmol) in a 250 mL Schlenk flask. Following the addition of glacial acetic acid (80 mL), the mixture was vigorously stirred for 60 min and refluxed at 130 °C for 12 h, followed by the removal of acetic acid via rotary evaporation. The product was then washed three times with a mixture of water and methanol (v/v, 9:1) and dried to yield a brown powder.

### Preparation of 5‐Carboxy‐1‐(d‐sulfobutyl)‐2,3,3‐trimethyl‐3H‐indolium Betaine (BTICA)

Accurately weighed 1,4‐butane sultone (7.89 g, 57.36 mmol) and TICA (2 g, 9.84 mmol), then added to a 100 mL Schlenk flask. After adding 1,2‐dichlorobenzene (40 mL), the mixture was thoroughly stirred and heated to reflux conditions overnight. Once the reaction was complete, the resulting precipitate was filtered, washed thoroughly three times with acetone, and subsequently dried to yield a red solid product.

### Preparation of ICG

Glutacondianil hydrochloride (0.81 g, 2.80 mmol) and BTICA (2 g, 5.80 mmol) were weighed and placed in a Schlenk flask containing a mixture of glacial acetic acid (18 mL) and acetic anhydride (30 mL). Then sodium acetate (0.83 g, 10 mmol) was added while stirring vigorously, and the suspension was heated to reflux conditions for 45 min. Afterward, the mixture was cooled and precipitated into cold diethyl ether, washed three times with diethyl ether, and recrystallized in a water/1‐propanol mixture (v/v, 1:4) at 4 °C overnight. The crystals were filtered, washed with cold 1‐propanol and cold diethyl ether, and dried under vacuum to yield a constant weight of green solid.

### Preparation of Nalpha‐Fmoc‐L‐lysine Hydrochloride Conjugated ICG (ICG‐lys‐FMOC)

Initially, the carboxyl group of ICG was activated to yield ICG‐NHS. In a 25 mL Schlenk flask under an argon atmosphere, 0.3 g (0.39 mmol) of ICG, 0.14 g (1.20 mmol) of NHS, 0.23 g (1.20 mmol) of EDC•HCl, and 0.015 g (0.12 mmol) of DMAP were dissolved in 10 mL of anhydrous DMSO and stirred for 30 h. The reaction mixture was then precipitated in cold acetone, filtered, washed, and vacuum‐dried to obtain ICG‐NHS. Subsequently, 300 mg (0.31 mmol) of ICG‐NHS and 300 mg (0.74 mmol) of nalpha‐Fmoc‐L‐lysine hydrochloride were dissolved in anhydrous DMSO (10 mL) and stirred for 5 h. Upon completion of the reaction, the product was precipitated, filtered, and thoroughly washed, followed by vacuum‐drying to obtain ICG‐lys‐FMOC as the final product.

### Preparation of Diethylenetriaminepentaacetic Acid Conjugated ICG‐lys (ICG‐lys‐DTPA)

Initially, the carboxyl group of DTPAA was activated to form DTPAA‐NHS, employing a method similar to that used for the preparation of ICG‐NHS. Subsequently, the protective group of ICG‐lys‐FMOC was removed by dissolving ICG‐lys‐FMOC (500 mg, 0.34 mmol) in anhydrous DMSO (8 mL) and adding 2 mL of piperidine (20%). Following a 30 min stirring period, the solution was dialyzed (1000 Da, MW cut‐off) in methanol for 24 h, followed by precipitation in diethyl ether. The resulting product was then filtered, washed thoroughly, and vacuum‐dried to yield a dark green ICG‐lys powder. Subsequently, DTPAA‐NHS (50 mg, 0.11 mmol) was dissolved in NaHCO_3_ (10 mL, 0.1 M) and vortexed for a period of 2 h in order to facilitate the hydrolysis of the anhydride group into a carboxylic acid. Then, ICG‐lys (50 mg, 0.049 mmol) was added, and the resulting solution was stirred for 6 h, followed by dialysis (500 Da, MW cut‐off) in water for 72 h and lyophilization, which produced a green solid of ICG‐lys‐DTPA.

### Preparation of ICG‐lys‐DTPA@Gd (ILD) and c(RGDyK)‐ICG‐lys‐DTPA@Gd (cRGD‐ILD)

The ICG‐lys‐DTPA molecule was initially employed for chelating the gadolinium ion. Specifically, 20 mg (0.011 mmol) of ICG‐lys‐DTPA was dissolved in 10 mL of PBS and mixed with 10 mg (0.027 mmol) of GdCl_3_•6H_2_O. After stirring for 24 h, the mixture was dialyzed (500 Da, MW cut‐off) against water for 72 h and lyophilized to obtain the non‐targeting probe ILD.

Additionally, 20 mg (0.0096 mmol) of ILD was redissolved in PBS and activated by stirring with 2.4 mg (0.021 mmol) of NHS, 0.4 mg (0.0029 mmol) of DMAP, and 4.1 mg (0.021 mmol) of EDC•HCl for 2 h. Then, 11.9 mg (0.0192 mmol) of c(RGDyK) peptide was added and stirred for 12 h. The mixture was dialyzed (500 Da, MW cut‐off) against water for 72 h and lyophilized to yield the targeting probe cRGD‐ILD.

### Molecular Probes Characterization

The ^1^H NMR spectra were conducted utilizing a Varian Unity 400 MHz Spectrometer (Bruker, Switzerland) in a variety of deuterated solvents. ESI‐MS were obtained with a Thermo Scientific LTQ XL mass spectrometer (Thermo, USA). UV absorption spectra were measured by a Unico UV‐2000 UV–vis Spectrophotometer (Shanghai, China). Photoluminescence excitation and emission spectra were recorded using an FSP920‐combined Time Resolved and Steady State Fluorescence Spectrometer (Edinburgh, UK), equipped with a 450‐W xenon lamp. Both the UV and fluorescence spectrophotometers were scanned within the wavelength range of 650–850 nm.

The generation of ROS was quantified using DPBF as a probe. Specifically, 50 µL of DPBF (1000 µg mL^−1^) was mixed with 3 mL of cRGD‐ILD or ILD (*C*_ICG_ = 10 µg mL^−1^) in methanol and exposed to an 808 nm laser (1.0 W cm^−2^) in a dark environment for different periods. The gradual decrease in the UV absorption of DPBF was monitored using a UV–vis spectrophotometer, and the correlation between the UV absorbance values and the laser exposure time was recorded.

The MRI sensitivity of cRGD‐ILD and ILD probes was evaluated on a 3.0 T Ingenia MR system provided by the First Hospital of Sun Yat‐sen University by measuring their *T*_1_ relaxation times. cRGD‐ILD and ILD were dissolved in PBS at predetermined concentrations in a 96‐well detachable plate. An inversion recovery spin echo sequence was executed with the parameters: repetition time (TR) = 5.27 ms, echo time (TE) = 2.29 ms, field of view (FOV) = 160 mm × 160 mm, acquisition matrix = 336 × 336, acquisition voxel = 0.48 mm × 0.48 mm × 2 mm, slice thickness = 2 mm, reconstruction matrix = 672 × 672, reconstruction voxel = 0.24 mm × 0.24 mm × 2 mm, number of flip angles = 4 (3°, 6°, 9°, 13°), and NSA = 1. Regions of interest (ROI, average size 10 mm^2^) were analyzed to acquire the *T_1_* relaxation times. The *r*_1_ was then calculated based on the slope of the linear plot between 1/*T_1_* and Gd concentration, determined through linear least‐squares regression analysis.

Furthermore, a fast spin echo *T*_1_‐weighted imaging protocol was optimized for sensitivity and resolution. The acquisition parameters were as follow:TR = 400 ms; TE = 13 ms; FOV = 80 mm × 80 mm; acquisition matrix = 192 × 192; voxel = 0.42 mm × 0.42 mm × 1 mm; slice thickness = 1 mm, reconstruction matrix = 384 × 384 × 2, reconstructed voxel = 0.24 mm × 0.24 mm × 1 mm; flip angle, 150°; and NSA = 8.

Additionally, the potential of molecular probes for PAI was evaluated. Solutions of cRGD‐ILD with various concentrations (*C*_ICG_ = 10, 20, 30, 40, 60, 80 µg mL^−1^), dissolved in PBS, were placed in a capillary tube and subjected to PAI using a TomoWave LOIS‐3D series photoacoustic imaging system (TomoWave Laboratories, Inc., USA). Image processing and subsequent data analysis were carried out utilizing the LOIS View 3D software.

Finally, cRGD‐ILD or ILD dissolved in PBS (*C*_ICG_ = 50 µg mL^−1^) was irradiated (808 nm, 1.0 W cm^−^^2^, 5 min) to assess temperature profiles. Subsequently, a digital thermographic camera (Fluke Ti27, IR Fusion Technology, USA) was used to capture infrared thermal images and measure temperatures. The relationship between temperature values and laser irradiation duration was recorded using AnalyzIR software (4.0.0). Furthermore, by controlling the laser on/off cycles, real‐time temperature changes were monitored during heating and cooling phases of both the cRGD‐ILD solution (*C*_ICG_ = 50 µg mL^−1^) and PBS control. The PCE (η) was subsequently calculated based on these measurements.^[^ ^41^ ^]^ Additionally, the photothermal heating curve of the solutions that comprised cRGD‐ILD and ILD molecular probes, dissolved in PBS, was assessed using a consistent method after subjecting them to laser irradiation (808 nm, 1.0 W cm^−^^2^, 5 min) at varying ICG concentrations ranging from 5 to 100 µg mL^−1^.

### Isolation of Endometrial Cells and Cell Culture

The Ishikawa cells were procured from Zhejiang Meisen Cell Technology Co. Ltd. (CTCC‐003‐0095, Zhejiang, China) and were cultivated in DMEM medium, enriched with 10% FBS and 1% penicillin/streptomycin, within a humidified incubator maintained at 37 °C with 5% CO_2_.

Endometrial specimens were prospectively acquired from April 2024 to April 2025 at our institution. The study cohort comprised 15 premenopausal women with surgically and histopathologically verified EMS, from whom paired ectopic and eutopic endometrial tissues were obtained during indicated hysteroscopic and laparoscopic procedures. Comparator normal endometrial samples (n = 5) were collected from controls undergoing surgery for benign gynecologic conditions unrelated to EMS. All patient specimens were collected with informed consent, and this research was approved by the Ethics Committee of the Sixth Affiliated Hospital of Sun Yat‐sen University (Ethics Code: 2024ZSLYEC‐206).

After PBS cleaning and impurity removal, tissues were minced and digested with 2.5 mg mL^−1^ type I collagenase (1:5) for 60–90 min in a 37 °C, 5% CO_2_ incubator. Digestion was stopped with DMEM/F12 medium, and the suspension was filtered through 70 and 40 µm sieves to separate epithelial cells and ESCs. The filtered liquid was centrifuged, and the ESC‐rich sediment was cultured in DMEM/F12 medium with 10% FBS and 1% penicillin/streptomycin at 37 °C in a humidified, 5% CO_2_ incubator. All experimental cells were in logarithmic growth phase.

### Cell Identification

NESCs from non‐EMS women and EuESCs and EESCs from EMS patients were plated in a 12‐well culture plate with a cell culture slide, grown to 40%–50% confluence, rinsed three times with PBS, and fixed with 4% paraformaldehyde for 15 min. The cells were added with 0.5% Triton X‐100 for 20 min to permeabilize the membranes, blocked with normal goat serum at room temperature for 1 h, and then incubated overnight at 4 °C with 300 µL of diluted primary antibodies (vimentin, 1:200, AF7013, Affinity, USA; cytokeratin, 1:300, 10712‐1‐AP, Proteintech, Beijing, China). Slides were then incubated with a FITC‐conjugated goat anti‐rabbit IgG secondary antibody (1:60, F0382, Sigma–Aldrich, USA) at room temperature for 1 h. Following incubation, nuclei were counterstained with DAPI for 5 min, and slides were mounted using an anti‐fade mounting medium. Finally, images were acquired using a CLSM (LSM 710, Carl Zeiss, Germany).

### Immunofluorescence Detection of ITGB3

The cells were seeded in 24‐well plates containing cell culture slides at a density of 1 × 10⁴ cells per well. Following adherence, samples were fixed with ice‐cold methanol (−20 °C) for 15 min, washed thoroughly with PBS, and blocked with 5% BSA at room temperature. Immunostaining was initiated by incubating slides overnight at 4 °C with a primary mouse monoclonal antibody anti‐ITGB3 (1:100, ab7166, Abcam, UK) on an orbital shaker. Subsequently, slides were incubated with FITC‐conjugated secondary goat anti‐mouse IgG (1:200, S0007, Affinity Biosciences, USA) for 60 min. Nuclear counterstaining was performed using DAPI‐containing mounting medium (Mounting Medium with DAPI‐Aqueous, Fluoroshield, ab104139, Abcam, UK). Fluorescent signals were visualized and captured using a CLSM. Image processing and analysis were conducted using ImageJ 1.53e (National Institutes of Health).

### Cellular Internalization of Probes

The internalization of ILD and cRGD‐ILD by cells was observed using CLSM, flow cytometry, and MRI. For visualization through CLSM, the cells were seeded onto confocal dishes at a density of 1 × 10^4^ cells per dish and allowed to incubate overnight at 37 °C. After incubation periods of 30 min, 1 h, and 2 h with molecular probes (*C*_ICG_ = 20 µg mL^−1^), the cells were fixed using 4% paraformaldehyde for 10 min. Subsequently, the cell nuclei were stained with a DAPI solution for 5 min. Following staining, the intracellular fluorescence emitted by the ICG was analyzed under a CLSM.

Furthermore, the cellular internalization of the probes was quantitatively evaluated using flow cytometry. The cells (1 × 10^4^ per well) were seeded in 24‐well plates, grown to 80%–90% confluence, and incubated with either cRGD‐ILD or ILD (*C*_ICG_ = 2.5 µg mL^−1^) for durations of 1 and 4 h. After these incubation periods, the cells were washed three times with PBS, trypsinized, and collected. The harvested cells were then analyzed using an Attune NxT flow cytometer (Invitrogen, USA). The data obtained was analyzed utilizing NovoExpress 1.4.0 software (Agilent, USA).

Cellular uptake of molecular probes in Ishikawa cells and EESCs was assessed via in vitro MRI. Cells (1 × 10^5^ per well) were seeded in 6‐well plates, cultured overnight at 37 °C, and incubated with probes at specified Gd concentrations for 2 h. After washing and trypsinization, cells were resuspended in 250 µL 1% agarose gel and loaded into 96‐well plates. MRI scans were conducted on a 3.0 T MR scanner, employing a fast spin echo *T*_1_‐weighted imaging protocol optimized for sensitivity and resolution. The acquisition parameters were as follow:TR = 350 ms; TE = 13 ms; FOV = 150 mm × 150 mm; acquisition matrix = 288 × 288; acquisition voxel = 0.52 mm × 0.52 mm × 1 mm; reconstruction matrix = 576 × 576; reconstruction voxel = 0. 26 mm × 0.26 mm × 1 mm; slice thickness, 1 mm; flip angle, 150°; and NSA = 1.

Additionally, an inversion recovery spin echo sequence was executed with the parameters: TR = 5.79 ms; TE = 2.37 ms; FOV = 160 mm × 160 mm; acquisition matrix 208 × 208; acquisition voxel = 0.77 mm × 0.77 mm × 1 mm; reconstruction matrix = 416 × 416; reconstruction voxel = 0.38 mm × 0.38 mm; slice thickness = 1 mm; number of flip angles = 4 (3°, 6°, 9°, 13°); and NSA = 1.

### Intracellular ROS Generation

DCFH‐DA was utilized to evaluate the intracellular ROS production induced by molecular probes under laser irradiation. Specifically, the cells (1 × 10^4^ per well) were seeded onto confocal dishes and incubated overnight at 37 °C. After incubation with cRGD‐ILD or ILD (*C*_ICG_ = 20 µg mL^−1^) for 4 h, 1 mL of DCFH‐DA (10 µM in non‐FBS medium) was added to the dishes, and the Ishikawa cells and EESCs were further incubated for 15 min. Following this, the cells were carefully rinsed three times with fresh PBS. Subsequently, the cells were irradiated with an 808 nm laser (1.0 W cm^−^^2^, 5 min). Intracellular ROS levels were then visualized and analyzed using a CLSM.

Additionally, a quantitative assessment of intracellular ROS production was performed using flow cytometry under the same conditions as the above experimental conditions. The cells were seeded in a 24‐well plate and treated with cRGD‐ILD or ILD (*C*_ICG_ = 20 µg mL^−1^), followed by staining with DCFH‐DA. The cells were then trypsinized and harvested. After exposure to an 808 nm laser (1.0 W cm^−^^2^, 5 min), the collected cells were analyzed on an Attune NxT flow cytometer (Invitrogen, USA). The resulting data was analyzed with NovoExpress 1.4.0 software (Agilent, USA).

### Cytotoxicity Experiment

The CCK‐8 assay was used to quantitatively assess the viability of Ishikawa cells and EESCs after treatment with cRGD‐ILD or ILD. The cells (1 × 10^4^ per well) were seeded in a 96‐well plate, allowed to adhere overnight, and then added to various concentrations of ICG (ranging from 10 to 300 µg mL^−1^) in the form of ILD and cRGD‐ILD. After 24 h, the medium was replaced with fresh medium containing 10% CCK‐8 reagent and incubated for an additional 2 h. The absorbance at 450 nm was measured using a Synergy 2 multimode microplate reader (BioTek, USA), with the wells without cells serving as blanks. All experiments were performed in triplicate.

### In Vitro Photothermal/Photodynamic Therapy

Ishikawa cells and EESCs were seeded in a 96‐well plate at a density of 1 × 10^4^ cells per well and allowed to adhere overnight. Subsequently, the cells were incubated with cRGD‐ILD and ILD at ICG concentrations ranging from 2.5 to 50 µg mL^−1^ for 2 h at 37 °C. Following this incubation, the cells were irradiated with an 808 nm laser (1.0 W cm^−2^, 5 min). The viability of cells was measured using the CCK‐8 assay.

Furthermore, 1 × 10^4^ cells were cultivated in 24‐well plates and subjected to treatment with cRGD‐ILD and ILD, each at ICG concentrations of 50 and 100 µg mL^−1^, for a duration of 2 h. Next, the cells were exposed to an 808 nm laser irradiation at a power density of 1.0 W cm^−2^ for 5 min. Medium was changed, and incubation continued for 36 h after irradiation. Subsequently, the cells were trypsinized, centrifuged, and harvested for the assessment of apoptosis. Initially, the cells were rinsed with PBS and then processed in accordance with the standardized protocol provided by the Annexin V‐FITC/PI Apoptosis Detection Kit (E‐CK‐A211, Elabscience, Wuhan). The processed cells were then analyzed using an Attune NxT flow cytometer (Invitrogen, USA). For data interpretation, NovoExpress 1.4.0 software (Agilent, USA) was employed.

In a separate experiment, 1 × 10^4^ cells were seeded into confocal dishes and cultured overnight at 37 °C. After incubation with cRGD‐ILD and ILD (*C*_ICG_ = 50 µg mL^−1^) for 2 h, the cells in the confocal dishes were irradiated with an 808 nm laser at a power of 1.0 W cm^−2^ for 5 min. Moreover, the cells were stained with calcein‐AM/PI to detect live and dead cells under CLSM.

### Animal Model

All animals were brought from Zhejiang Viton Lihua Laboratory Animal Technology Co. The in vivo experiments were approved by the Laboratory Animal Ethics Committee of the Sixth Affiliated Hospital of Sun Yat‐sen University (Approval number: IACUC‐2023042701, IACUC‐2023101206). These animals were subjected to a tightly controlled environmental condition consisting of a temperature of 25 ± 2 °C and a 12 h light/12 h dark cycle, and the modeling was performed after 7 days of normal rearing.

Specific pathogen‐free Wistar female rats (6–8 weeks old, 160–200 g) received 0.5 mg kg^−1^ of β‐estradiol 17‐pentanoate by gavage daily for 3 days pre‐surgery. Rats were anesthetized by intraperitoneal injection of 1% sodium pentobarbital (45 mg kg^−1^). A 2 cm‐long uterus was excised after laparotomy and rinsed twice with saline. The uterus was incised longitudinally so that the mucosal side faced the peritoneum and the plasma side faced the abdominal cavity, and the uterine tissue was sutured to the abdominal wall in the left lower abdomen. Post‐surgery, rats were gavaged with 0.5 mg kg^−1^ β‐estradiol 17‐pentanoate every 4 days for 3 doses. After 4 weeks, the abdominal ectopic grafts were palpably enlarged and suitable for therapeutic and imaging applications.

As shown in previous references,^[^ ^19^ ^]^ to establish a xenograft EMS model in nude mice, female BALB/c nude mice (5‐6 weeks, 18‐20 g) served as recipients, and female Sprague–Dawley rats (7‐9 weeks, 200–240 g) with regular 4–5 days estrous cycles were selected as donors after a week of acclimatization. Prior to surgery, nude mice received daily intramuscular β‐estradiol 17‐pentanoate (0.25 µg g^−1^) for 3 days. The EMS model was induced by subcutaneous transplantation of ≈40 mg of rat uterine fragments into nude mice. The uterine horns from euthanized donors were sterilely collected, bisected, minced, and suspended in DMEM/F12 medium before injection. Nude mice then received β‐estradiol 17‐pentanoate (0.25 µg g^−1^) injections every 2 days, and endometriotic lesions reaching 100 mm^3^ were used for subsequent experiments.

### Detection of Integrin *α*_V_ and *β*_3_ in Endometriotic Lesions

Normal endometrial tissues and ectopic lesions from the EMS rat model were paraffin‐embedded, sectioned, and assayed for integrin *α*_v_ and *β*_3_ expression by immunohistochemistry. Sections were deparaffinized, antigen‐retrieved, and treated with 3% hydrogen peroxide for 25 min at room temperature to block endogenous peroxidase. They were then incubated with 3% BSA for 30 min and stained overnight at 4 °C with primary rabbit monoclonal antibodies anti‐integrin *α*_v_ (1:500, ab179475, Abcam, UK) or *β*_3_ (1:100, AF6086, Affinity, USA), respectively. After incubation with an HRP‐conjugated secondary antibody (1:200, GB23303, Servicebio, Wuhan, China) for 50 min, DAB staining and hematoxylin counterstaining of nuclei were performed for 3 min. Finally, sections were dehydrated, sealed, and viewed under a white light microscope. Image processing and analysis were conducted using ImageJ 1.53e.

Paraffin sections of ectopic endometrial tissues from ovarian endometrioma of premenopausal EMS patients and normal endometrium from patients without EMS undergoing laparoscopy and hysteroscopy were processed for immunofluorescence staining analysis. Sections underwent deparaffinization, antigenic repair, and blocking of endogenous peroxidase with 3% hydrogen peroxide. They were then incubated with 3% BSA, followed by overnight incubation with primary rabbit monoclonal antibodies anti‐integrin *α*_v_ (1:2500, ab179475, Abcam, UK). HRP‐labeled goat anti‐rabbit IgG (1:500, GB23303, Servicebio, Wuhan, China) and iF555‐Tyramide (1:500, G1223, Servicebio, Wuhan, China) were added sequentially. After another antigen repair, sections were incubated overnight with primary rabbit monoclonal antibodies anti‐integrin *β*_3_ (1:1000, AF6086, Affinity, USA), followed by HRP‐labeled goat anti‐rabbit IgG (1:500, GB23303, Servicebio, Wuhan, China) and iF488‐Tyramide (1:500, G1231, Servicebio, Wuhan, China). Nuclei were counterstained with DAPI, and sections were sealed with an anti‐fluorescence quenching sealer. Images were captured using a CLSM. Image processing and analysis were conducted using ImageJ 1.53e.

### In Vivo Infrared Thermography

To investigate the photothermal heating efficacy at lesion sites in EMS mice, in vivo infrared thermography studies were conducted. After modeling, the EMS mice (ICG, 500 µg kg^−1^) were intravenously administered with cRGD‐ILD, ILD, or PBS solutions via tail vein injection (n = 3). After a 1 h post‐injection interval, the mice were subjected to 808 nm laser irradiation (1.0 W cm^−^^2^, 5 min). Thermal responses were monitored in real‐time using a digital thermographic camera (Fluke Ti27, IR Fusion Technology, USA). Infrared thermal images were captured, and temperature measurements were recorded. The temporal correlation between temperature elevation and laser irradiation duration was analyzed using AnalyzIR software (4.0.0).

### In Vivo Fluorescence Imaging

In vivo fluorescence imaging studies were conducted to evaluate the biodistribution of molecular probes in EMS rats and mice. After modeling, EMS rats (ICG, 1000 µg kg^−1^) and mice (ICG, 500 µg kg^−1^) were injected with cRGD‐ILD or ILD solution via the tail vein (n = 3). Fluorescence acquisitions were then captured using an In Vivo Imaging System FX Pro (Carestream Health Inc., New Haven, CT, USA) at various time points before and after injection. Following euthanasia, the rats and mice were dissected, and their major organs (heart, liver, spleen, lungs, and kidneys) as well as the EMS lesions were harvested for ex vivo fluorescence imaging.

Additionally, to further evaluate the probe's off‐target effects and clearance data, three healthy BALB/c nude mice received an intravenous injection of cRGD‐ILD (ICG, 1000 µg kg^−1^). In vivo fluorescence imaging was conducted pre‐injection and at multiple time points post‐injection. The mice were sacrificed 10 days post‐injection, and major organs (heart, liver, spleen, lungs, and kidneys) were harvested for ex vivo fluorescence imaging.

### In Vivo MRI

To assess the in vivo MRI capabilities of cRGD‐ILD compared to ILD, MRI scans were performed on rats 28 days after modeling. Prior to the imaging procedure, the rats were anesthetized through intraperitoneal injection of 1% sodium pentobarbital at a dosage of 45 mg kg^−1^. Following anesthesia, the rats were positioned inside a 3.0 T MRI scanner sourced from the First Hospital of Sun Yat‐sen University.

Prior to drug administration, the rats underwent *T*_1_‐weighted imaging using the fast spin‐echo sequence with the following parameters: TR = 400 ms; TE = 12 ms; FOV = 80 mm × 80 mm; matrix size = 672 × 672; voxel size = 0.24 mm × 0.24 mm; slice thickness = 1 mm; flip angle = 150°; and NSA = 5.

Subsequently, the rats were injected via the tail vein with either cRGD‐ILD or ILD at a standard dose of 4.5 mg Gd per kg of body weight. *T*_1_‐weighted imaging using the fast spin‐echo sequence was repeated at 30 min and 5 h post‐injection, respectively, with adjusted parameters: TR = 425 ms; TE = 12 ms; FOV = 80 mm × 80 mm; matrix size = 384 × 384; voxel size = 0.42 mm × 0.42 mm; slice thickness = 1 mm; flip angle = 150°; and NSA = 7.

### In Vivo PAI

BALB/c nude mice with EMS lesions were randomly assigned to either the ILD or cRGD‐ILD group (n = 3). Within each group, three mice were intravenously injected with equivalent amounts of ILD and cRGD‐ILD (ICG, 4 µg g^−1^). PAI was conducted using a TomoWave LOIS‐3D series photoacoustic imaging system before injection and at 30 min, 5 h, 24 h, and 48 h post‐injection. Photoacoustic image processing and analysis were conducted using LOIS View 3D software and ImageJ 1.53e. ROIs were manually delineated on axial photoacoustic images to quantify lesion signal intensity (SI). Normalized photoacoustic intensity was calculated as SI_post_/SI_pre_, where SI_pre_ represents baseline intensity prior to intravenous injection, and SI_post_ denotes intensity measured at serial timepoints following administration of ILD or cRGD‐ILD solution.

### In Vivo Anti‐EMS Efficacy

After 21 days of endometrial tissue transplantation, nude mice were randomly divided into six groups (n = 5) receiving treatments of PBS, NIR laser irradiation (laser), ILD, cRGD‐ILD, ILD plus NIR laser irradiation (ILD + laser), and cRGD‐ILD plus NIR laser irradiation (cRGD‐ILD + laser). Mice were injected with different formulations via the tail vein every 3 days until euthanasia, with an ICG dose of 3 mg kg^−1^. For groups receiving NIR laser irradiation, the procedure was conducted 1 h post‐administration with a power of 1.0 W cm^−^^2^ for 10 min. Mouse body weights were recorded every 3 days, and xenograft growth was monitored using a vernier caliper. EMS rats (n = 4) underwent the same treatment strategy after 28 days of autotransplantation, with body weight changes monitored every 3 days. After 18 days of treatment, rats were euthanized for autograft size measurement. Nude mice were euthanized after 18 days for graft extraction, followed by H&E staining and TUNEL assay. Briefly, sections were deparaffinized, hydrated, stained with H&E, and visualized under an optical microscope.

TUNEL staining was performed following the instructions provided in the FITC TUNEL Cell Apoptosis Detection Kit (G1501, Servicebio, Wuhan). Paraffin sections were dewaxed, hydrated, repaired with proteinase K, and permeabilized with Triton X‐100. After incubating with buffer at room temperature for 10 min, a reaction solution containing TdT enzyme, dUTP, and buffer (1:5:50 ratio) was added and incubated at 37 °C for 1 h. Nuclei were counterstained with DAPI for 10 min, followed by three 5 min PBS washes (pH 7.4) on a decolorizing shaker. The slides were then blocked with an anti‐fluorescence quenching blocker (G1401, Sevier, Wuhan). The slides were visualized under CLSM with DAPI (blue light, excited at 330–380 nm, emitted at 420 nm) and FITC (green light, excited at 465–495 nm, emitted at 515–555 nm).

Furthermore, major organs (heart, liver, spleen, lungs, kidneys, and uterus) were excised for H&E staining. Blood was collected via the eye socket, and serum was obtained by letting the blood clot at room temperature for 1 h in a standard blood collection tube, followed by centrifugation at 3000 g for 15 min at 4 °C. The supernatant was stored at −80 °C for future liver and kidney function tests. Routine blood tests were conducted using a Myriad Veterinary Automatic Blood Cell Analyzer (China), while serum samples were analyzed with an Automatic Biochemical Analyzer (China) to measure AST, ALT, Cr, and BUN levels.

### Statistical Analysis

The results were presented as mean values ± standard deviation. Statistical analyses were performed using GraphPad Prism 6 software (GraphPad Software, Inc., La Jolla, CA, USA). Comparisons among groups were assessed using one‐way analysis of variance (ANOVA) or two‐tailed Student's *t*‐test, with statistical significance set at *p* < 0.05. LSD post‐hoc test was used for the multirange test.

## Conflict of Interest

The authors declare no conflict of interest.

## Supporting information



Supporting Information

Supplemental Movie 1

Supplemental Movie 1

## Data Availability

The data that support the findings of this study are available from the corresponding author upon reasonable request.
