# Supplementary material for: A Multifunctional Molecular Probe for Multimodal Imaging‐Guided Potent Photothermal/Photodynamic Therapy of Endometriosis
Source: Adv Sci (Weinh). 2025 Aug 28;12(43):e11126. doi: 10.1002/advs.202511126 (PMC12631912; doi:10.1002/advs.202511126)
Supplement: Supplementary file 1 — Supporting Information [file ADVS-12-e11126-s001.docx]

*Supporting Information*

# A Multifunctional Molecular Probe for Multimodal Imaging-Guided Potent Photothermal/Photodynamic Therapy of Endometriosis

*Qiyu Zhong^1,2#^, Shuguang Yang^4#^, Xiao Li^1,2#^, Zhuang Jin^1,2^, Jianyu Ma^1,2^, Zhouzhou Liao^1,2^, Jinbo Li^1,2^, Bo Li^5^*, Xintao Shuai^3^*, Shuqin Chen^1,2^**

^1^Department of Gynecology, The Sixth Affiliated Hospital, Sun Yat-sen University, Guangzhou 510655, China

^2^Biomedical Innovation Center, The Sixth Affiliated Hospital, Sun Yat-sen University, Guangzhou 510655, China

^3^Nanomedicine Research Center, The Third Affiliated Hospital of Sun Yat-sen University, Guangzhou 510630, China

^4^Department of Orthopaedics, Shanghai Key Laboratory for Prevention and Treatment of Bone and Joint Diseases, Shanghai Institute of Traumatology and Orthopaedics, Ruijin Hospital, Shanghai Jiao Tong University School of Medicine, 197 Ruijin 2nd Road, Shanghai 200025, China

^5^Guangzhou Municipal and Guangdong Provincial Key Laboratory of Molecular Target & Clinical Pharmacology, the NMPA and State Key Laboratory of Respiratory Disease, School of Pharmaceutical Sciences, Guangzhou Medical University, Guangzhou 511436, China

^#^These authors contributed equally to this work.

***Corresponding authors**

Prof. Shuqin Chen, e-mail: chshqin@mail.sysu.edu.cn

Prof. Xintao Shuai, e-mail: shuaixt@mail.sysu.edu.cn

Prof. Bo Li, e-mail: libo@gzhmu.edu.cn

## Supplementary Figures


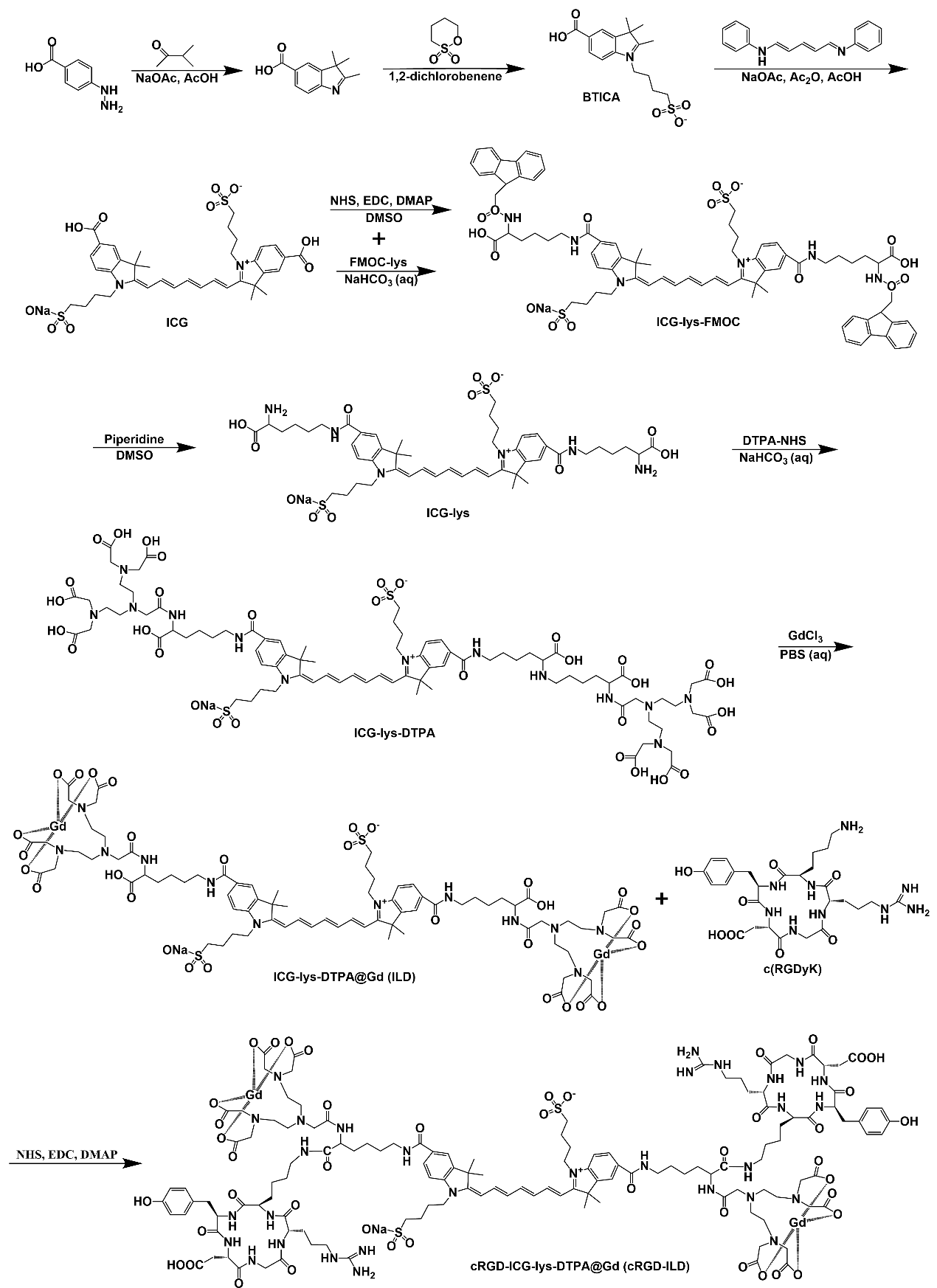


**Scheme S1.** A graphical illustration of the methodology employed in the fabrication of the water-soluble molecular probes ILD and cRGD-ILD.


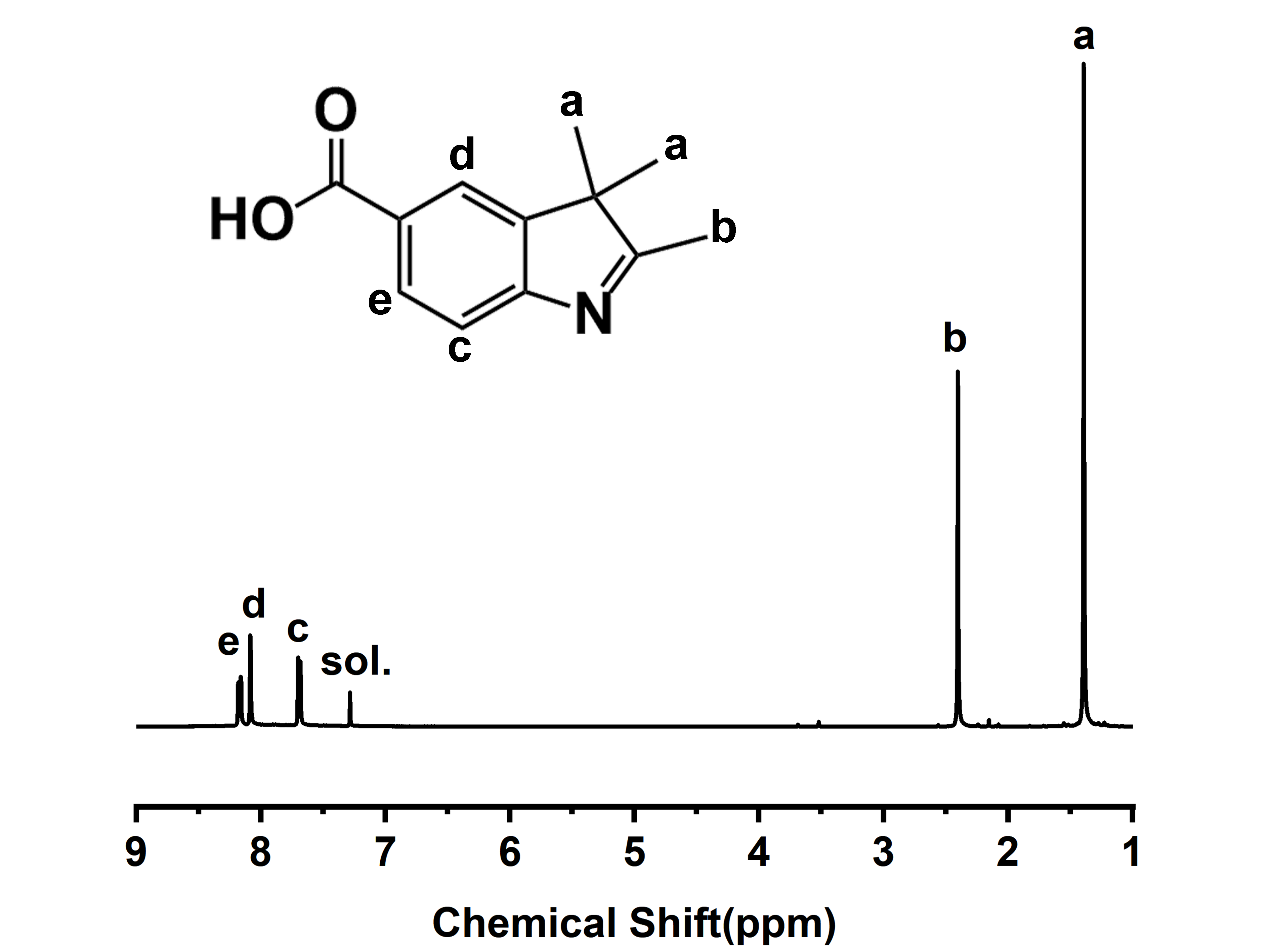


**Figure S1.** ^1^H NMR spectrum of TICA. ^1^H NMR (400 MHz, CDCl_3_, 298 K): δ (ppm) = 1.39 ppm (-C(C***H_3_***)_2_-, a), 2.40 ppm (-C(C***H_3_***) =N-, b), 7.70 ppm (-C***H***- of benzyl group, c), 8.09 ppm (-C***H***- of benzyl group, d), 8.15 ppm (-C***H***- of benzyl group, e).


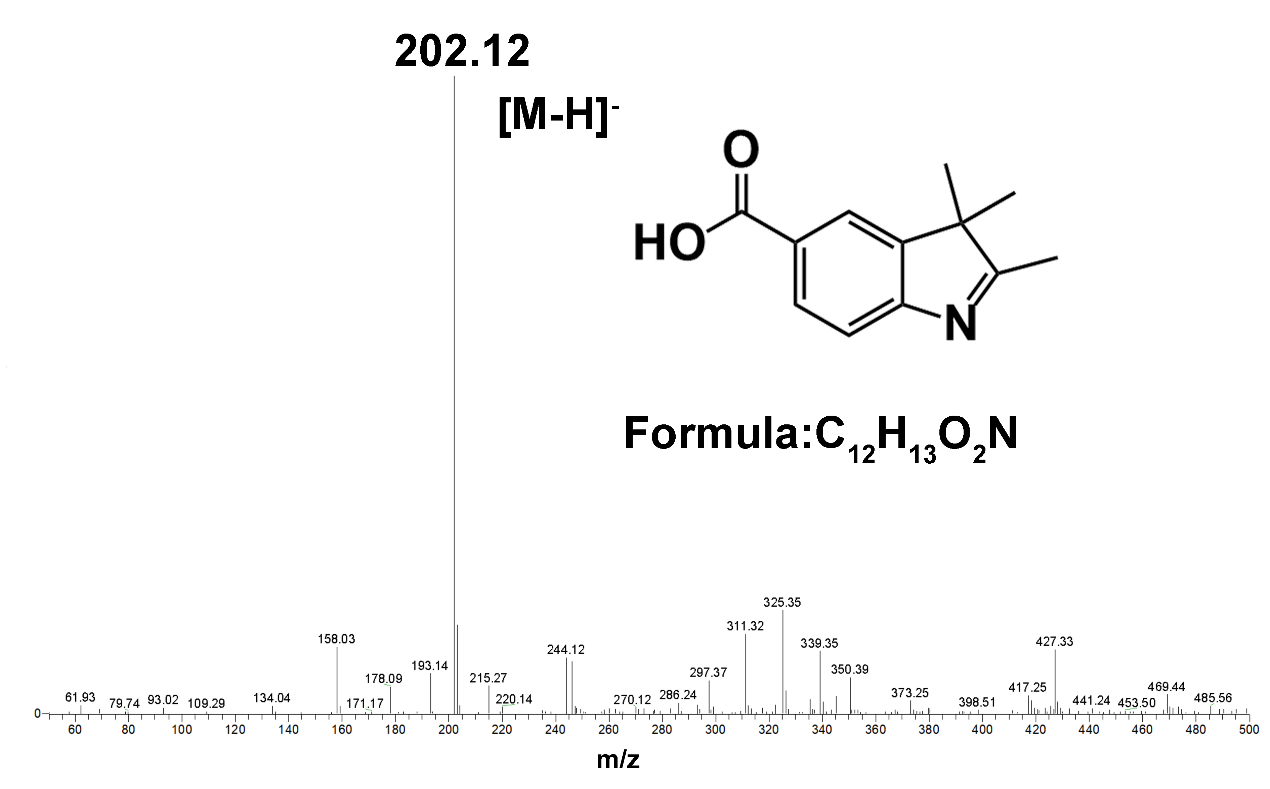


**Figure S2**. ESI mass spectrum of TICA.


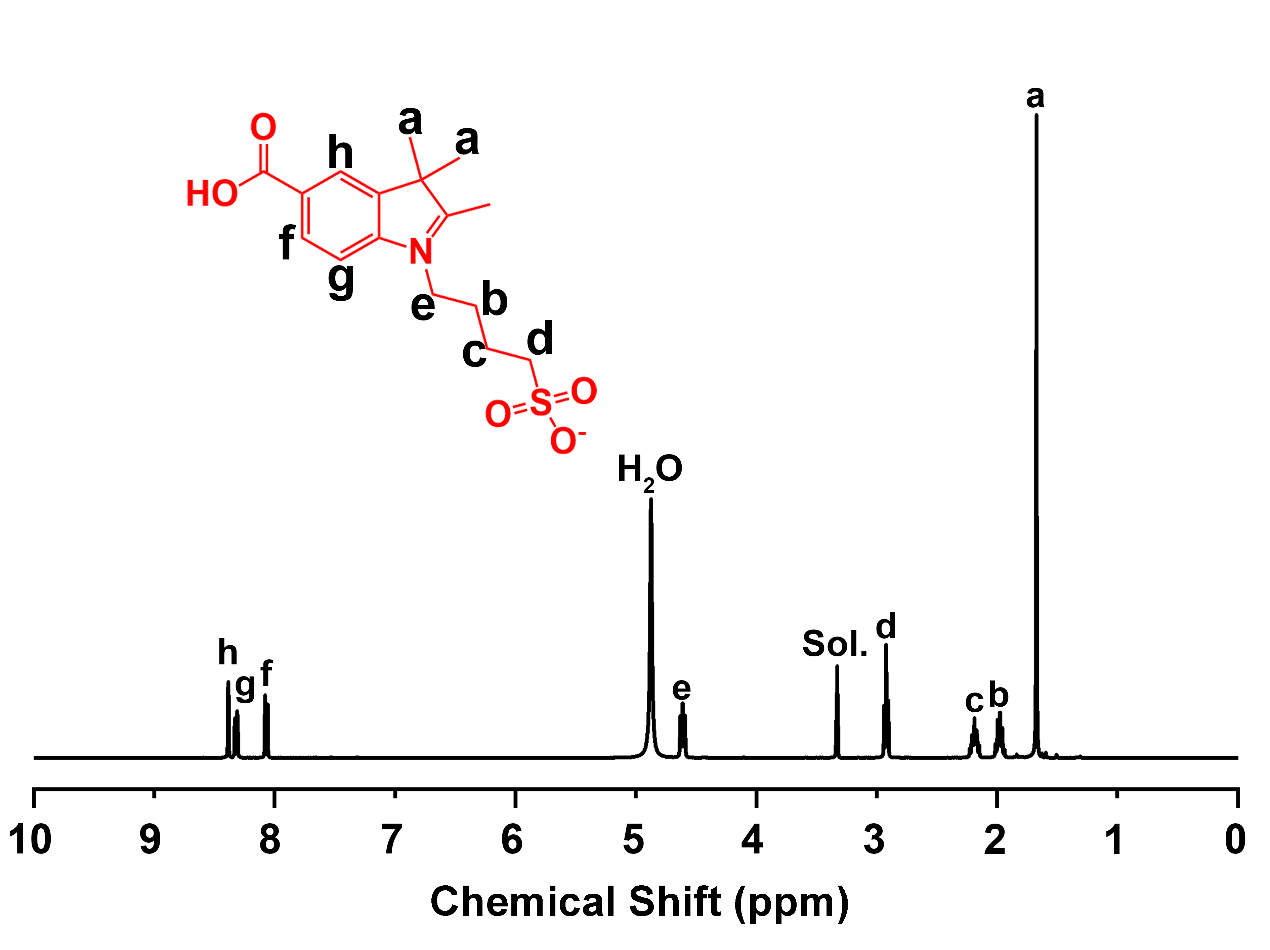


**Figure S3.** ^1^H NMR spectrum of BTICA. ^1^H NMR (400 MHz, MeOD, 298 K): δ (ppm) = 1.67 ppm (-C(C***H***_3_)_2_-, a), 1.98 ppm (-CH_2_C***H***_2_CH_2_CH_2_SO_3_-, b), 2.19 ppm (-CH_2_CH_2_C***H***_2_CH_2_SO_3_-, c), 2.92 ppm (-CH_2_CH_2_CH_2_C***H***_2_SO_3_-, d), 4.61 ppm (-C***H***_2_CH_2_CH_2_CH_2_SO_3_-, e), 8.08 ppm (-C***H***- of benzyl group, f), 8.33 ppm (-C***H***- of benzyl group, g), 8.39 ppm (-C***H***- of benzyl group, h).


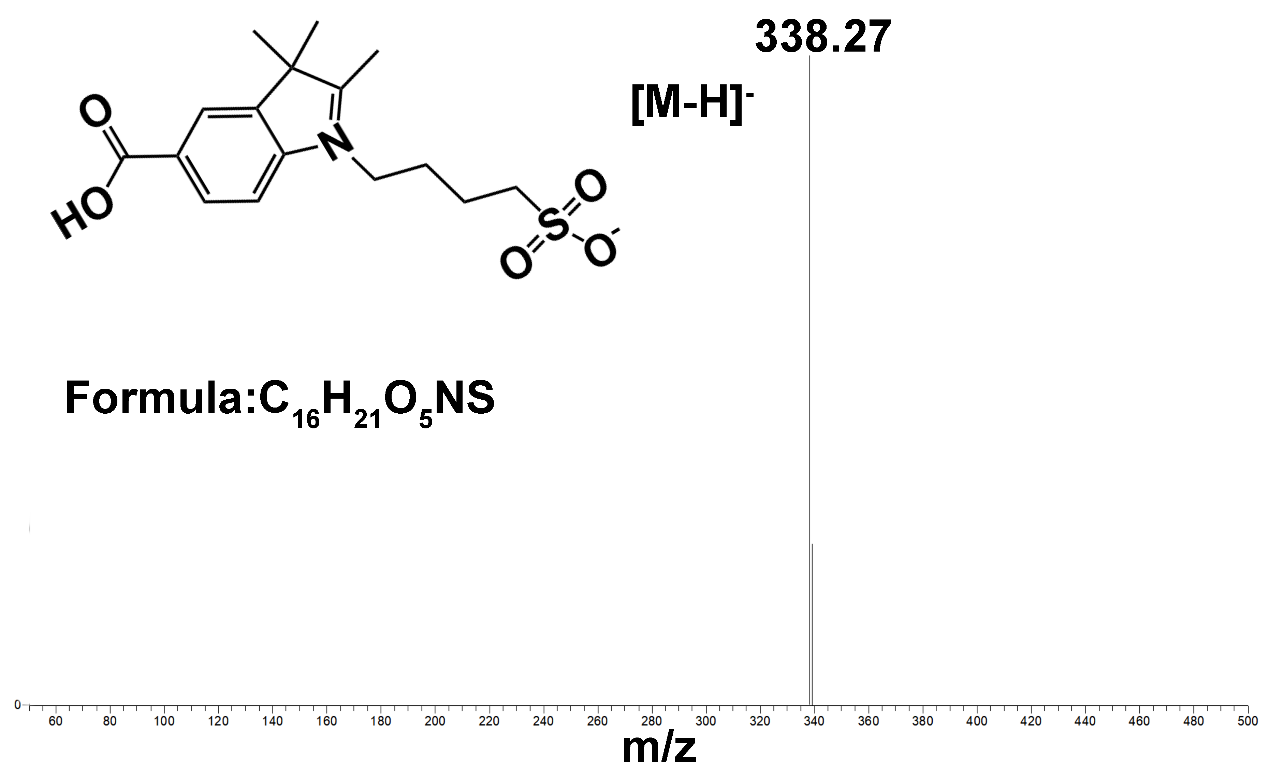


**Figure S4**. ESI mass spectrum of BTICA.


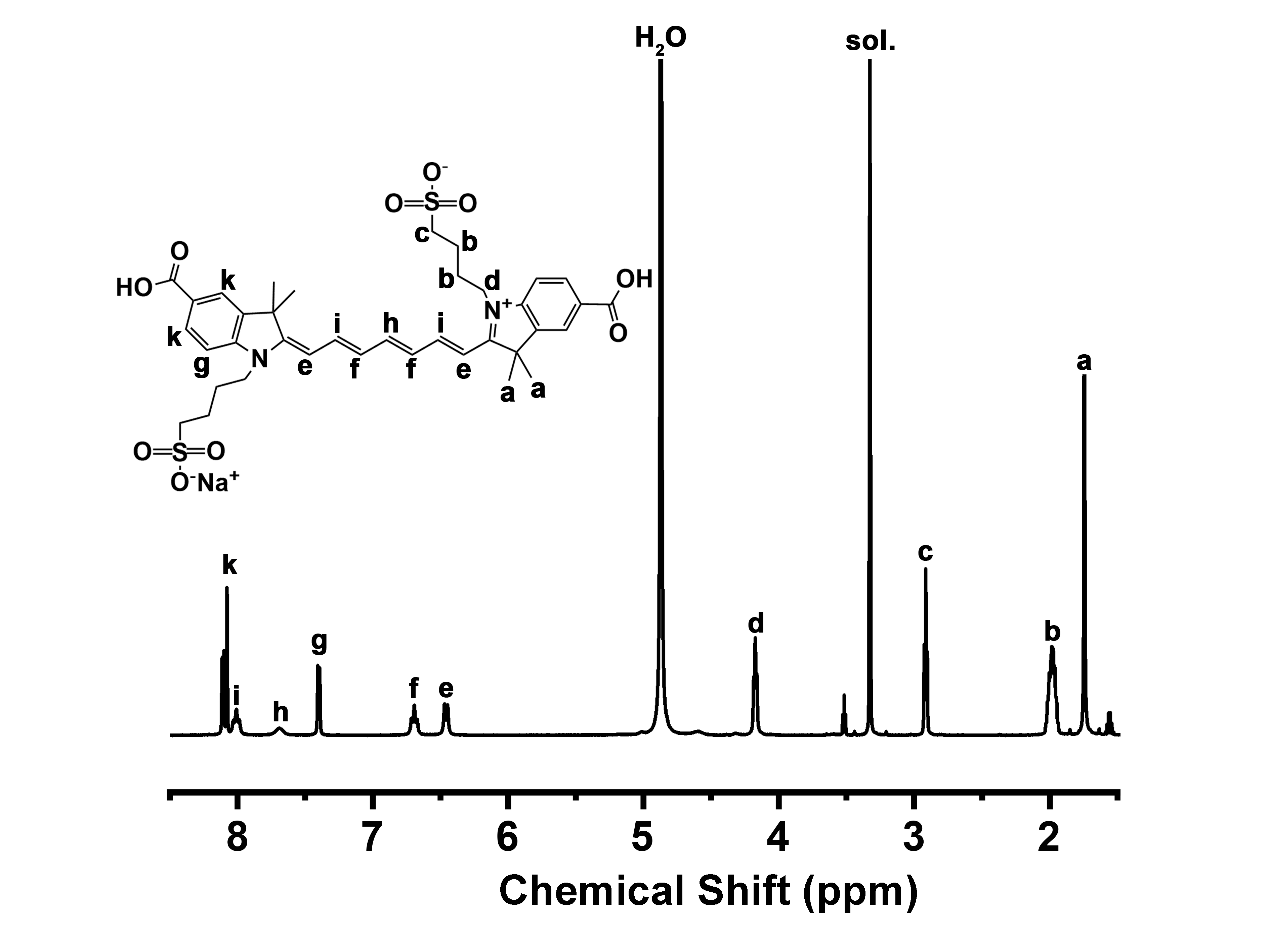


**Figure S5**. ^1^H NMR spectrum of ICG. ^1^H NMR (400 MHz, MeOD, 298 K) : δ (ppm) = 1.75 ppm (-C(C***H_3_***)_2_-, a), 1.98 ppm (-CH_2_C***H_2_***C***H_2_***CH_2_SO_3_-, b), 2.92 ppm (-CH_2_CH_2_CH_2_C***H_2_***SO_3_-, c), 4.17 ppm (-C***H_2_***CH_2_CH_2_CH_2_SO_3_-, d), 6.47 ppm (-C=C***H***-CH=CH-CH=CH-CH=C***H***-C-, e), 6.69 ppm (-C=CH-CH=C***H***-CH=C***H***-CH=CH-C-, f), 7.41 ppm (-C***H***- of benzyl group, g), 7.70 ppm (-C=CH-CH=CH-C***H***=CH-CH=CH-C-, h), 8.01 ppm (-C=CH-C***H***=CH-CH=CH-C***H***=CH-C-, i), 8.08 ppm (-C***H***- of benzyl group, k).


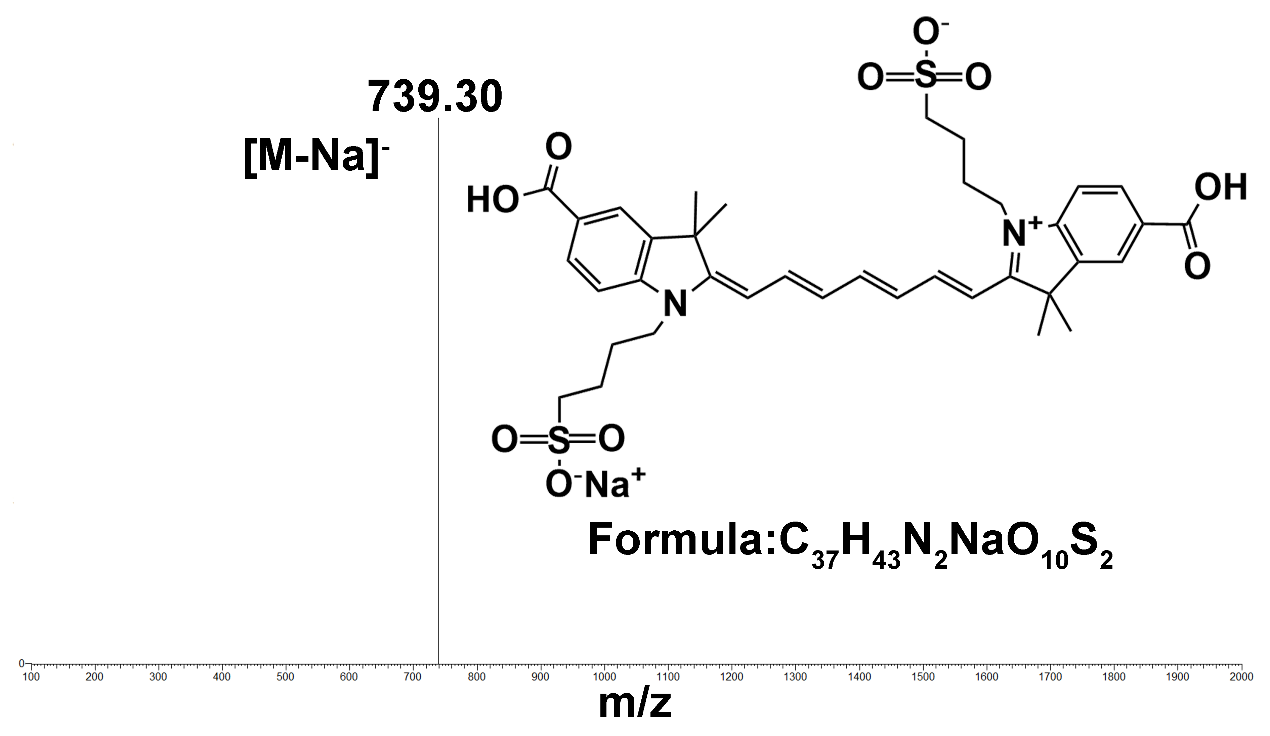


**Figure S6**. ESI mass spectrum of ICG.


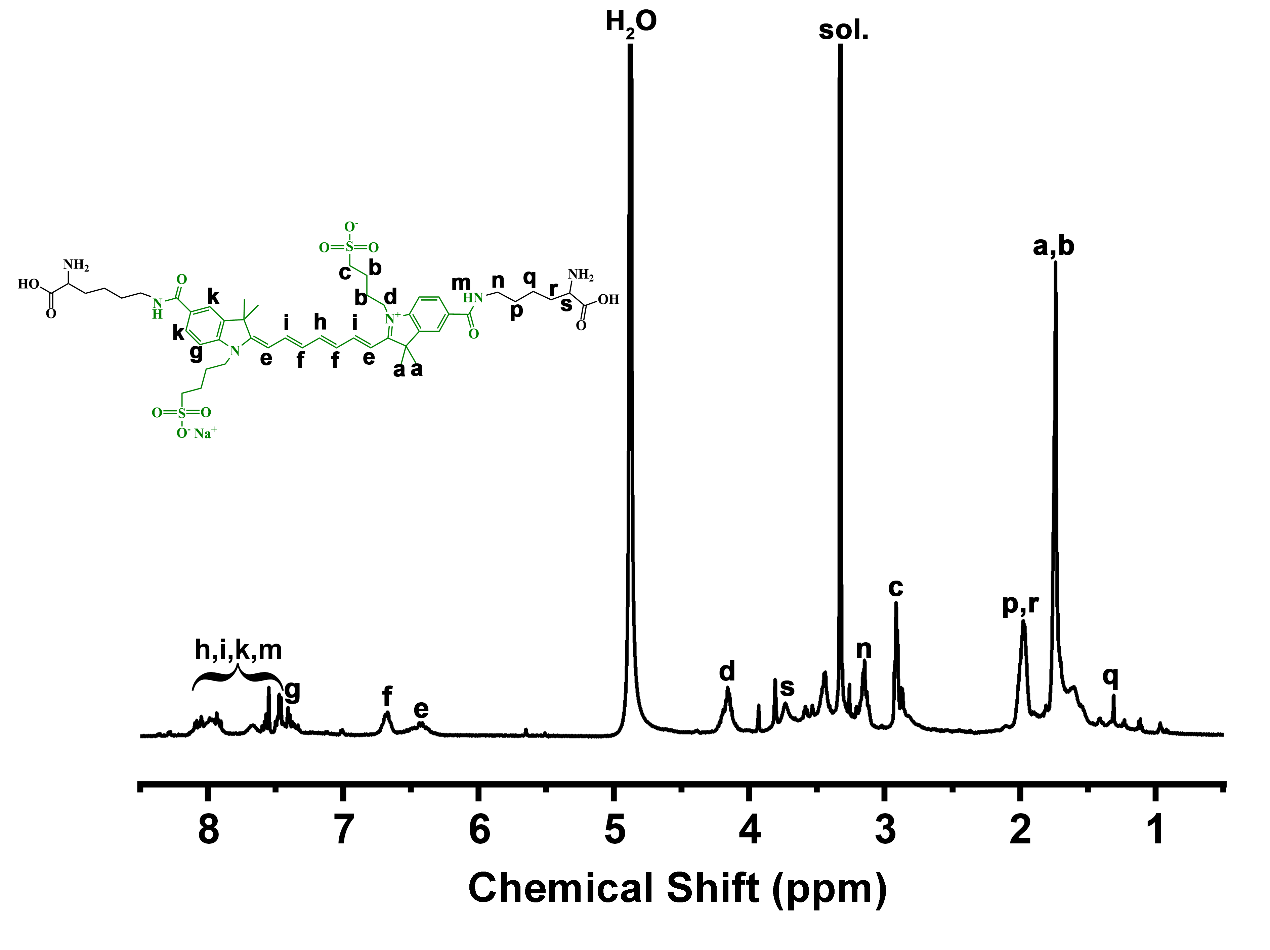
**Figure S7**. ^1^H NMR spectrum of ICG-lys. ^1^H NMR (400 MHz, MeOD, 298 K): δ (ppm) = 1.31 ppm (-NH-CH_2_CH_2_C***H_2_***CH_2_- of lys, q), 1.40-1.80 ppm (-C(C***H_3_***)_2_-, a; -CH_2_C***H_2_***C***H_2_***CH_2_SO_3_-, b), 1.81-2.10 ppm (-NH-CH_2_C***H_2_***CH_2_C***H_2_***- of lys, p, r), 2.91 ppm (-CH_2_CH_2_CH_2_C***H_2_***SO_3_-, c), 3.16 ppm (-NH-C***H_2_***CH_2_CH_2_CH_2_- of lys, n), 3.73 ppm (-C***H***(NH_2_)-COOH, s), 4.16 ppm (-C***H_2_***CH_2_CH_2_CH_2_SO_3_-, d), 6.41 ppm (-C=C***H***-CH=CH-CH=CH-CH=C***H***-C-, e), 6.68 ppm (-C=CH-CH=C***H***-CH=C***H***-CH=CH-C-, f), 7.40 ppm (-C***H***- of benzyl group, g), 7.43-8.27 ppm (-C=CH-CH=CH-C***H***=CH-CH=CH-C-, h; -C=CH-C***H***=CH-CH=CH-C***H***=CH-C-, i; -C***H***- of benzyl group, k; -CO-N***H***-(CH_2_)_4_-, m).


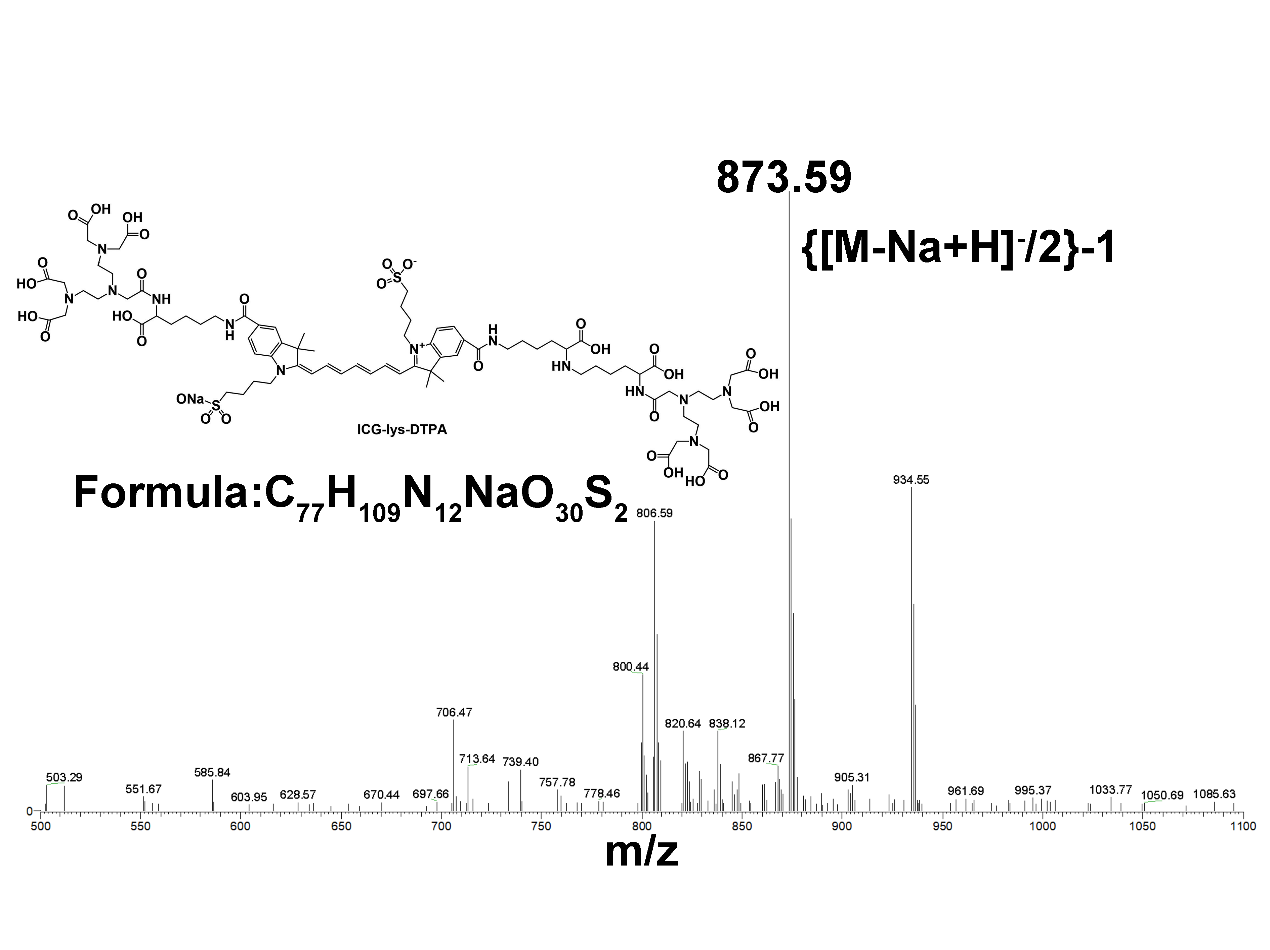


**Figure S8.** ESI mass spectrum of ICG-lys-DTPA.


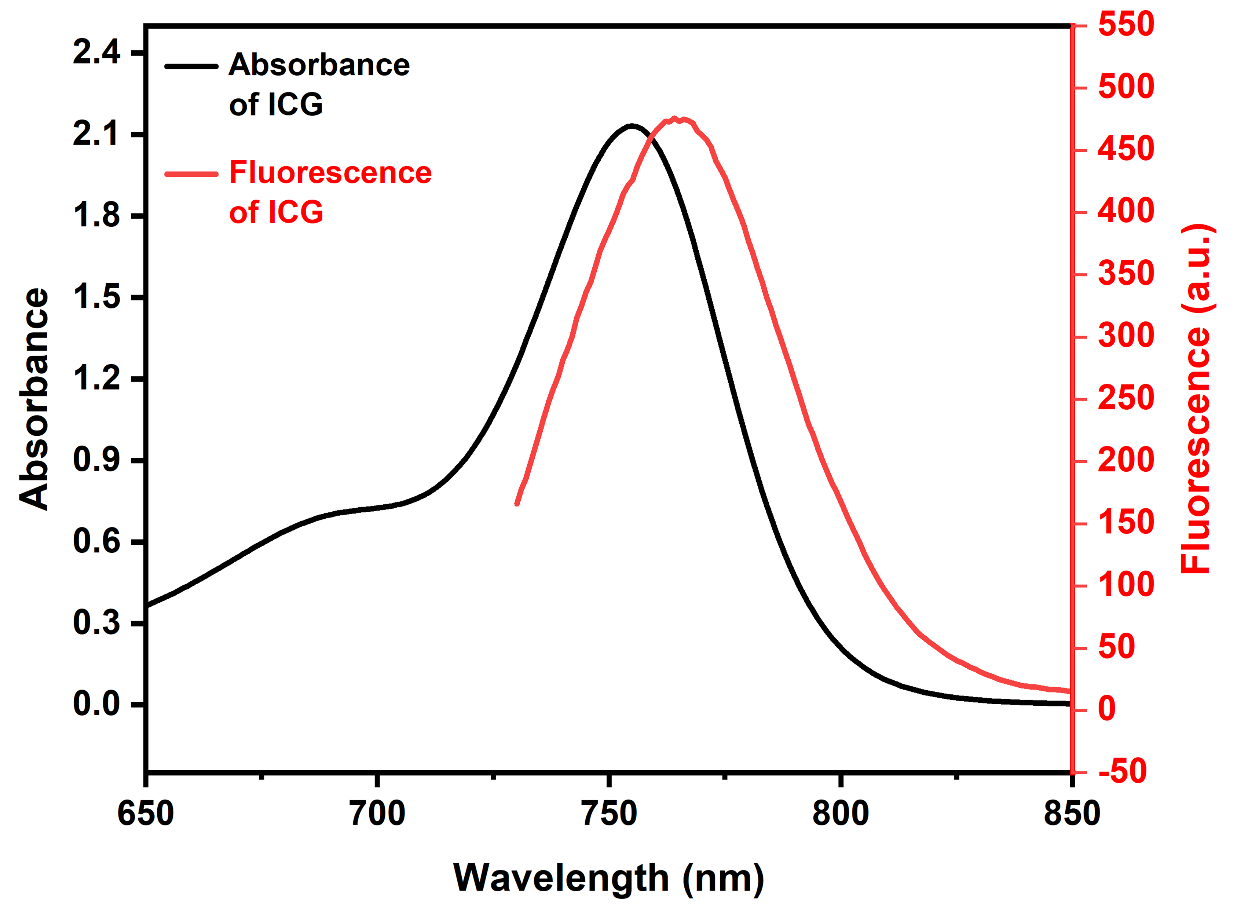


**Figure S9.** UV absorption spectrum and fluorescence emission spectrum (λ_ex_ = 755 nm) of ICG (*C*_ICG_ = 5 μg/mL) in PBS.


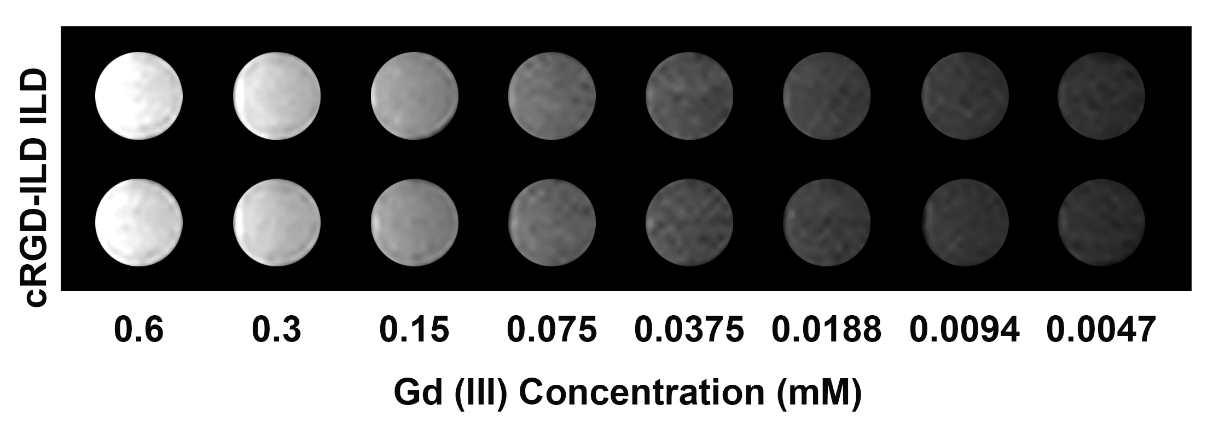


**Figure S10.** *T1*-weighted images of ILD and cRGD-ILD at various Gd (III) concentrations.


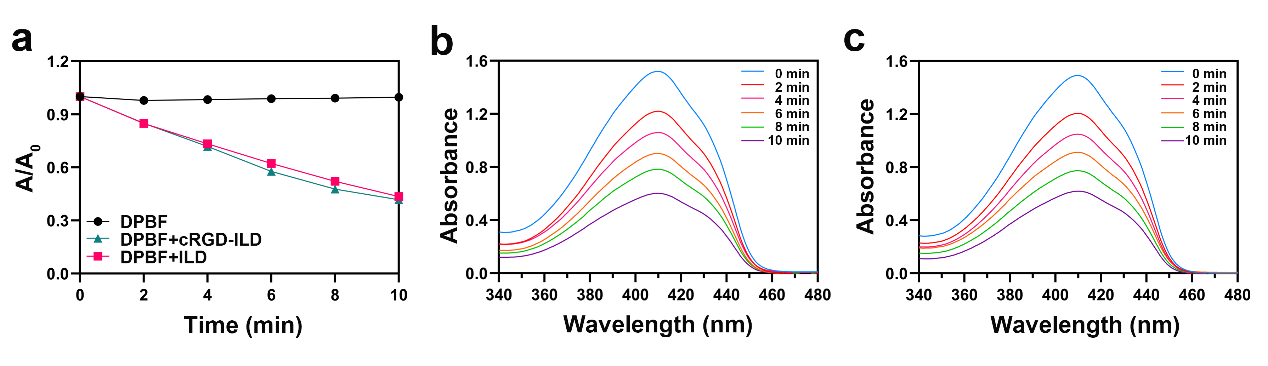


**Figure S11.** ROS detection of molecular probes. (a) Normalized absorbance at 410 nm of DPBF solutions (1000 µg/mL) containing cRGD-ILD or ILD (*C*_ICG_ = 10 µg/mL) under 808 nm laser irradiation (1.0 W/cm^2^, 10 min). Time-dependent DPBF absorbance changes with (b) cRGD-ILD or (c) ILD under identical irradiation conditions.


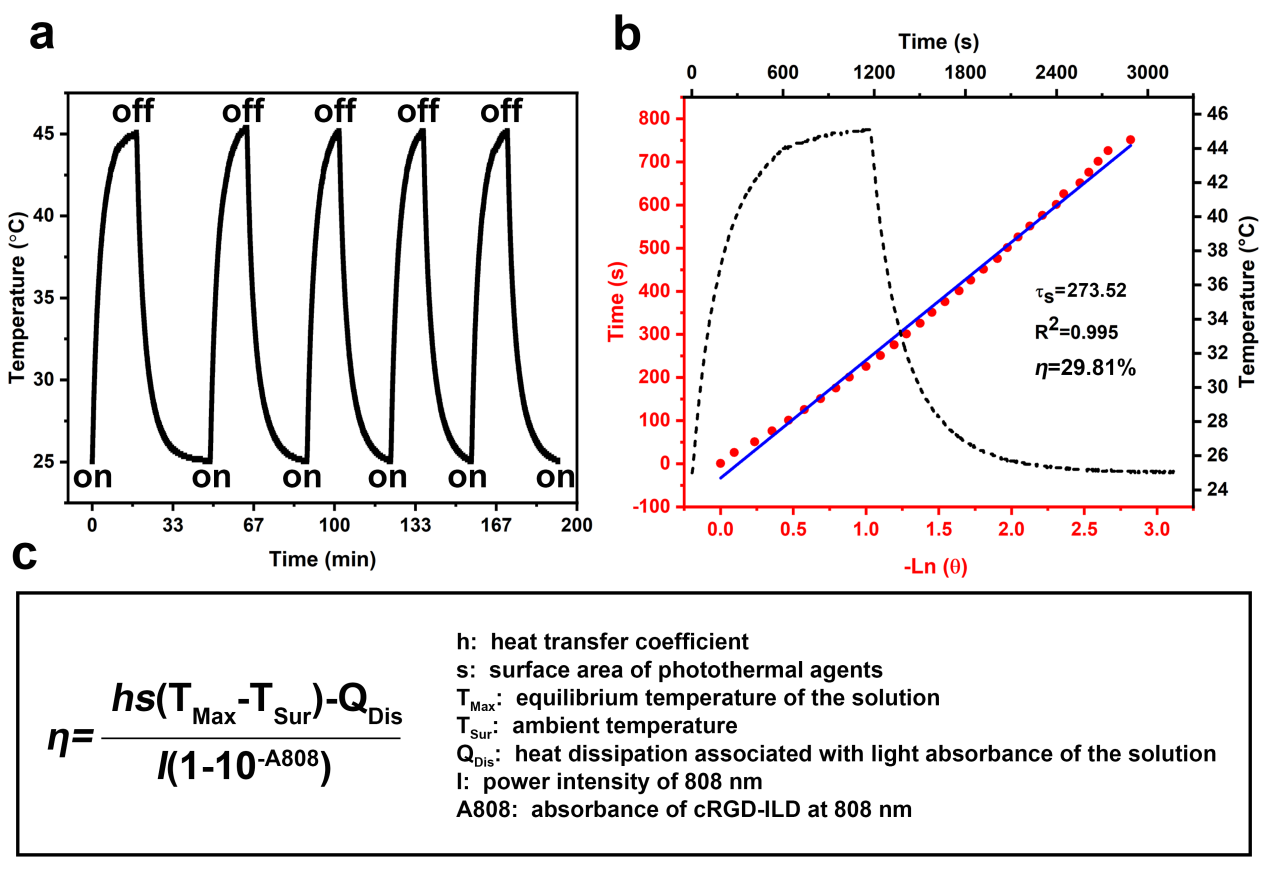


**Figure S12.** The photothermal testing of cRGD-ILD solution. (a) The photothermal stability of the cRGD-ILD solution (50 μg/mL, 1 W/cm²) was tested by controlling the photothermal switch over 5 photothermal cycles. (b) The heating and cooling curves of the cRGD-ILD solution (50 μg/mL, 1 W/cm²) and the linear fitting of cooling time versus -ln (ө). (c) The calculation formula for photothermal conversion efficiency and the meanings represented by each symbol.


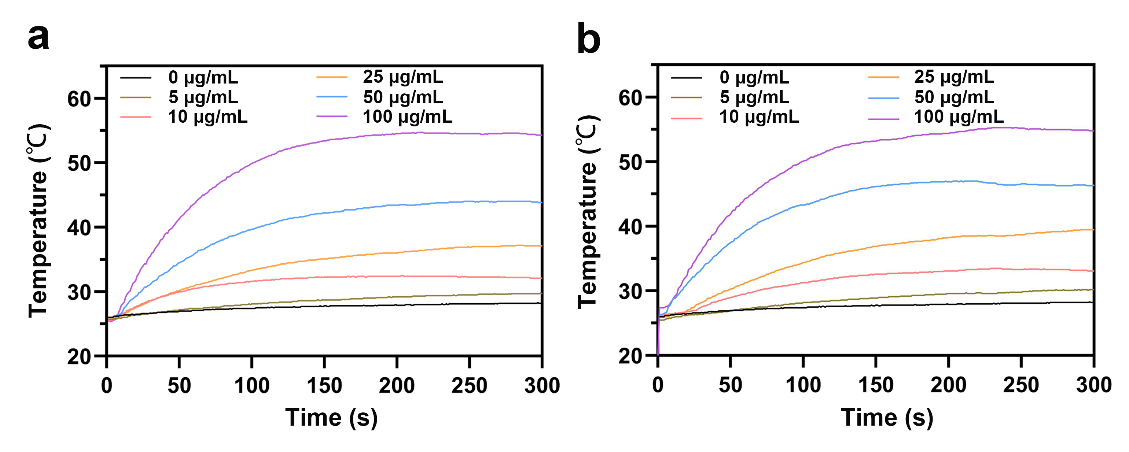


**Figure S13.** Photothermal conversion efficiency of molecular probes. (a) Temperature evolution profiles for cRGD-ILD and (b) ILD solutions at indicated concentrations under continuous 808 nm laser irradiation (1.0 W/cm², 5 min).


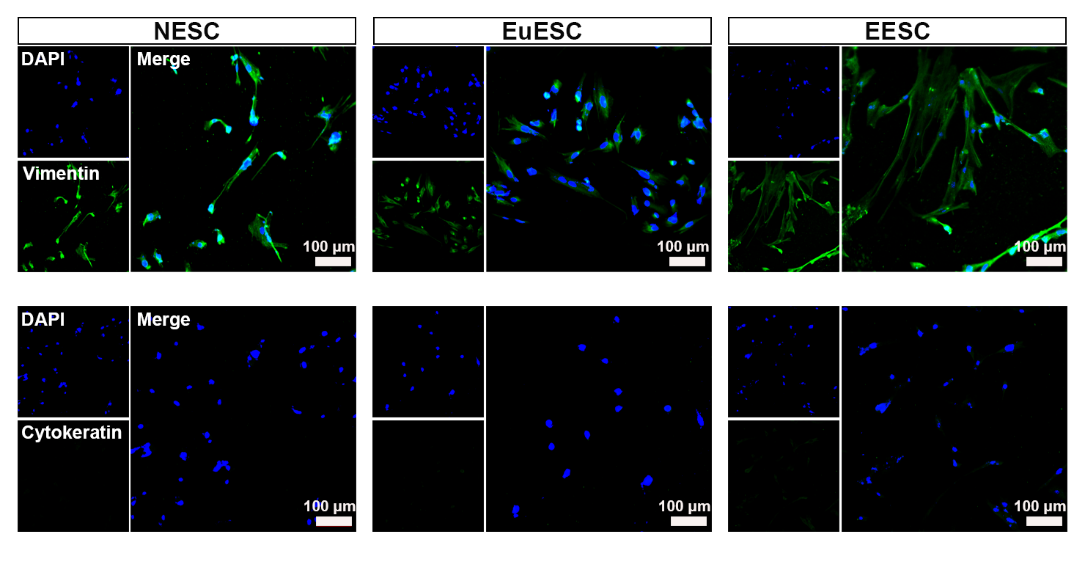


**Figure S14.** Fluorescence microscopy images showing vimentin (up, green fluorescence) and cytokeratin (down, green fluorescence) expression in normal endometrial stromal cells (NESCs), eutopic endometrial stromal cells (EuESCs), and ectopic endometrial stromal cells (EESCs). Nuclei are counterstained with DAPI (blue fluorescence).

**
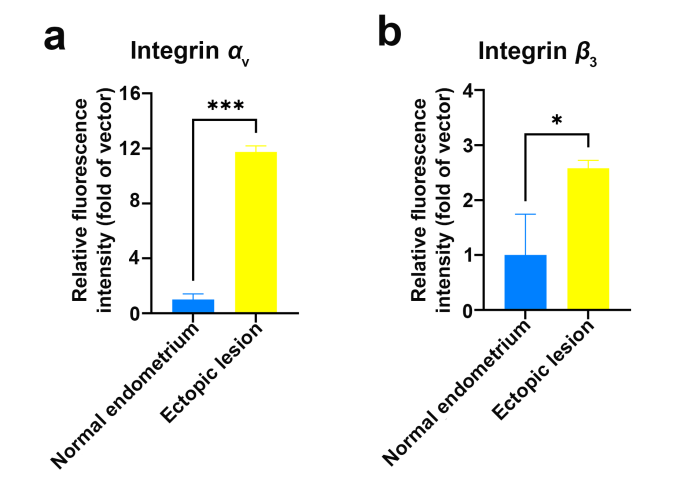
**

**Figure S15.** Quantification of integrin *α*_V_ (a) and *β*_3_ (b) expression based on immunofluorescence images in ectopic lesions from EMS patients and normal endometrium from non-EMS patients. Data are presented as mean ± SD, n = 3 per group. Statistical significance was determined by two-tailed unpaired Student's *t*-test; **P* < 0.05, ****P* < 0.001.


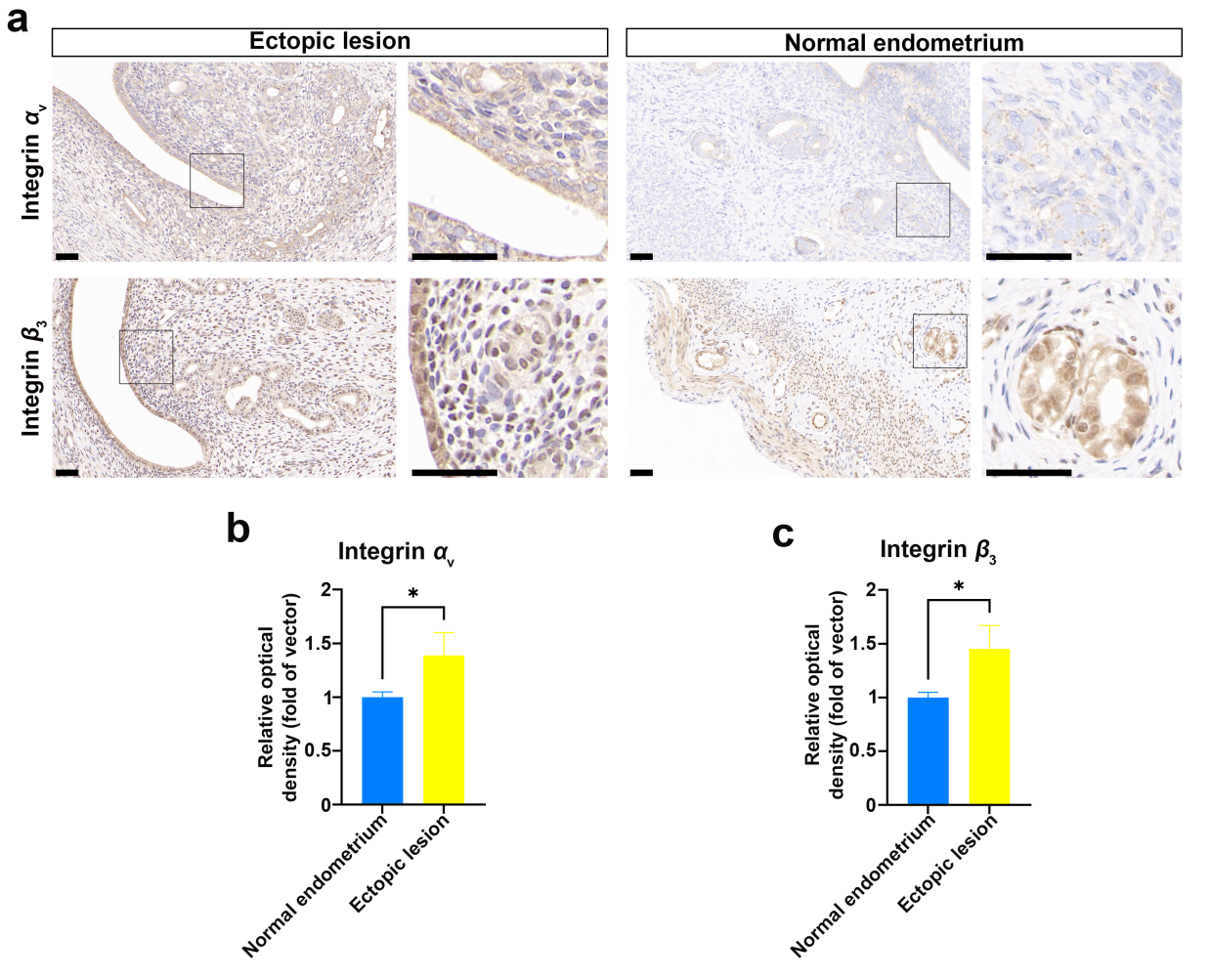


**Figure S16.** Expression levels of integrin *α*_V_ and *β*_3_ in EMS rat models. (a) Typical immunohistochemical staining of integrin *α*_V_ and *β*_3_ in ectopic lesions and normal endometrium from EMS rat models. Scale bar: 50 μm. (b, c) Quantification of integrin *α*_V_ (b) and *β*_3_ (c) expression levels from (a). Data are presented as mean ± SD, n = 3 per group. Statistical significance was determined by two-tailed unpaired Student's *t*-test; **P* < 0.05.


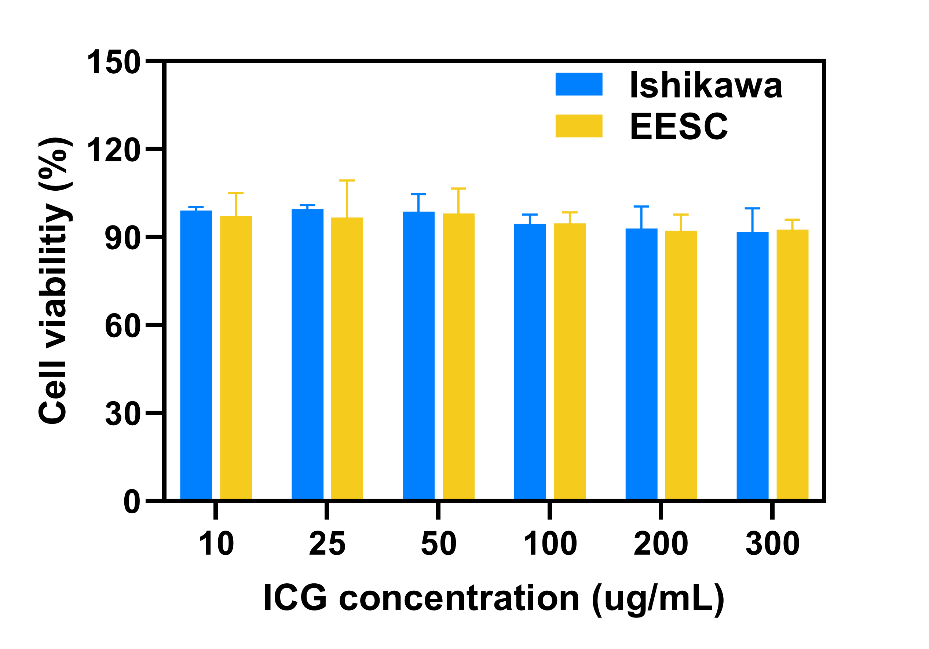


**Figure S17.** Cell viability of Ishikawa cells and EESCs after 24 h incubation with cRGD-ILD or ILD. Data are presented as mean ± SD, n = 4 independent cell samples per group.


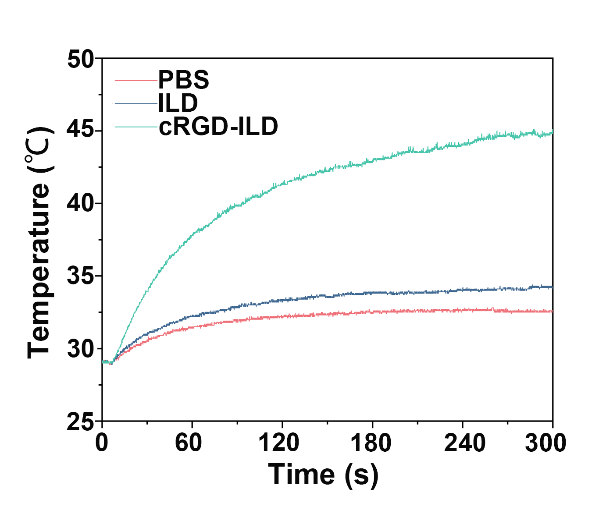


**Figure S18.** Photothermal temperature curves of EMS lesions irradiated with an 808 nm laser (1.0 W/cm², 5 min) at different time points post-injection of cRGD-ILD, ILD, or PBS.

**
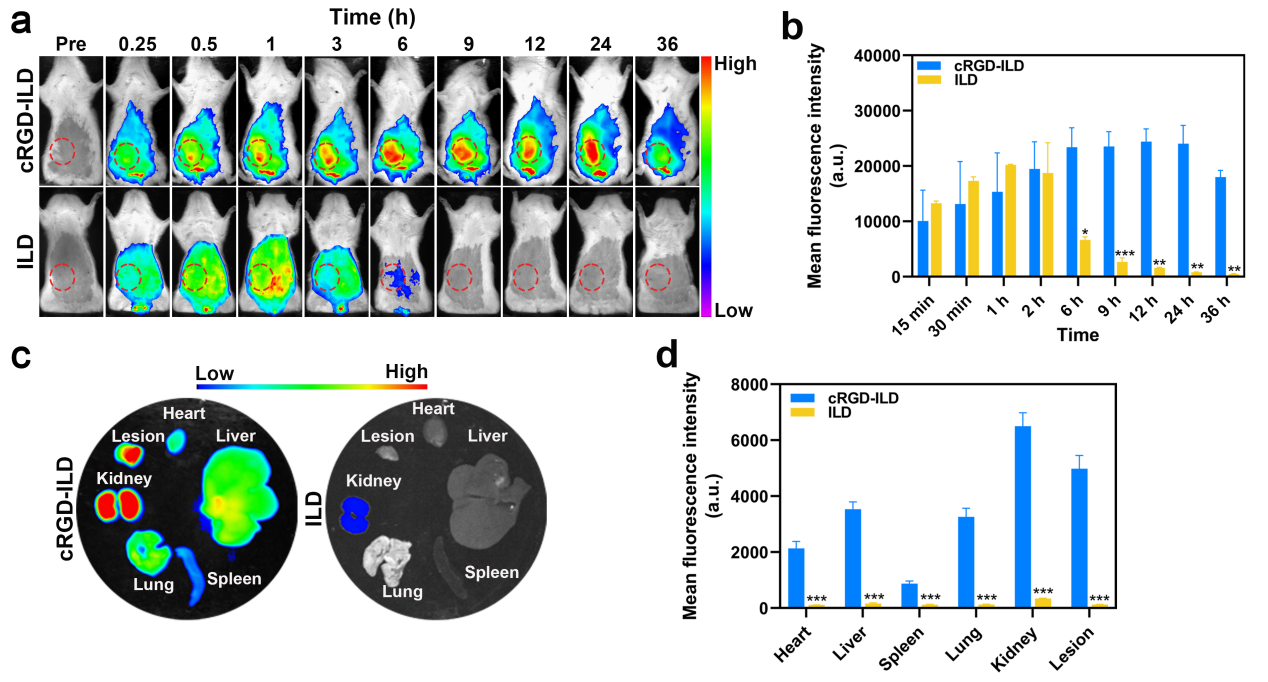
**

**Figure S19.** *In vivo* and *ex vivo* fluorescence imaging in rats with autologous EMS grafts. (a) *In vivo* fluorescence imaging of EMS rat post-injection of cRGD-ILD or ILD. Red circles denote lesion sites. (b) Time-dependent *in vivo* fluorescence intensities at ectopic lesion sites. (c) *Ex vivo* fluorescence imaging of ectopic lesions and major organs harvested from rats 36 h post-injection in (a). (d) *Ex vivo* fluorescence quantification of ectopic lesions and major organs (heart, liver, spleen, lung, kidney) across treatment groups. Data are presented as mean ± SD, n = 3 rats per group. Statistical signiﬁcance was calculated by a two-tailed unpaired Student’s *t*-test, **P* < 0.05, ***P* < 0.01, ****P* < 0.001 (compared with cRGD-ILD group).

**
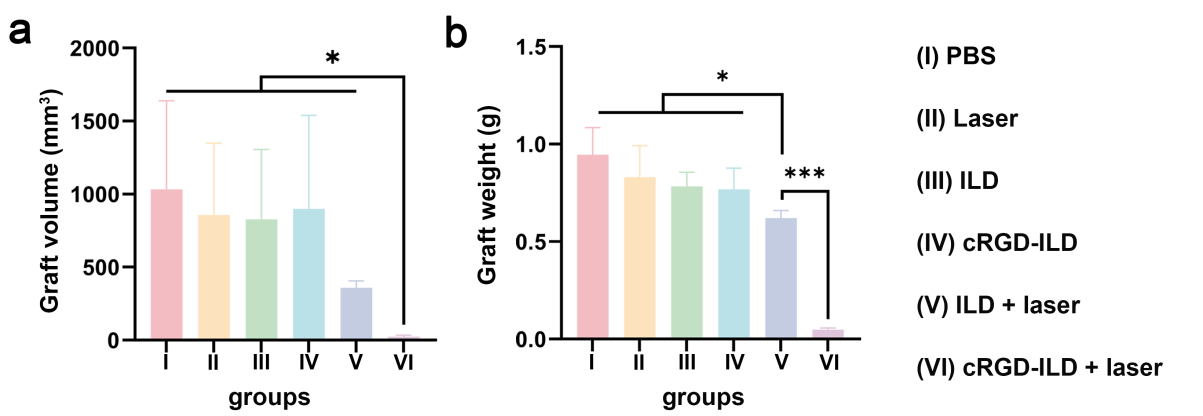
**

**Figure S20.** Therapeutic efficacy of molecular probes in autografted EMS rats. (a) Mean lesion volumes and (b) mean lesion weights across treatment groups. Data are presented as mean ± SD, n = 4 rats per group. Statistical signiﬁcance was calculated by a two-tailed unpaired Student’s *t*-test; **P* < 0.05, ****P* < 0.001.


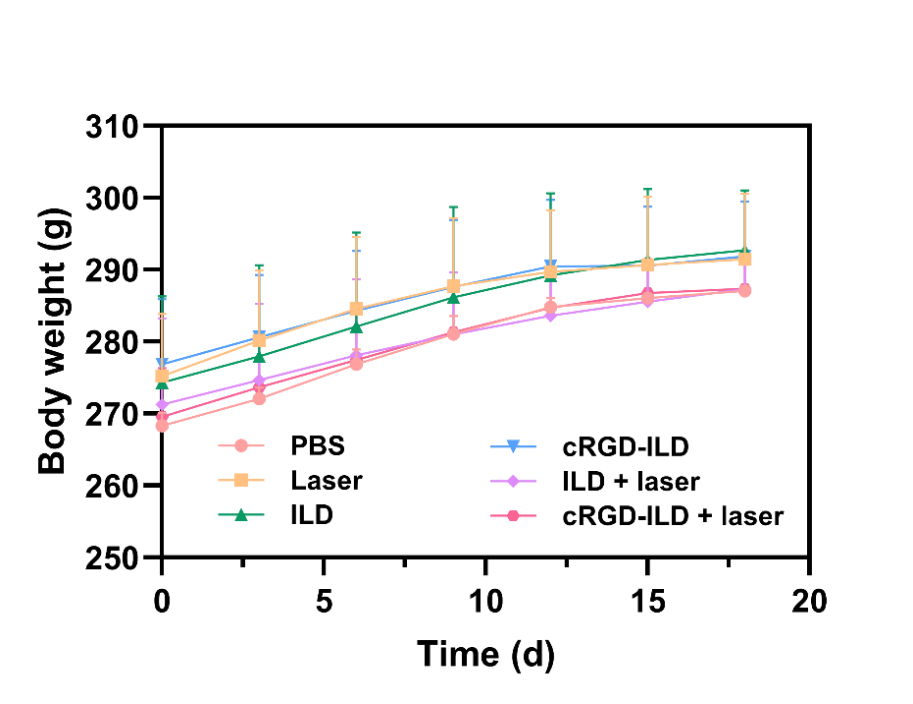


**Figure S21.** Body weight dynamics in EMS model rats during therapeutic intervention. Data are presented as mean ± SD, n = 4 rats per group.


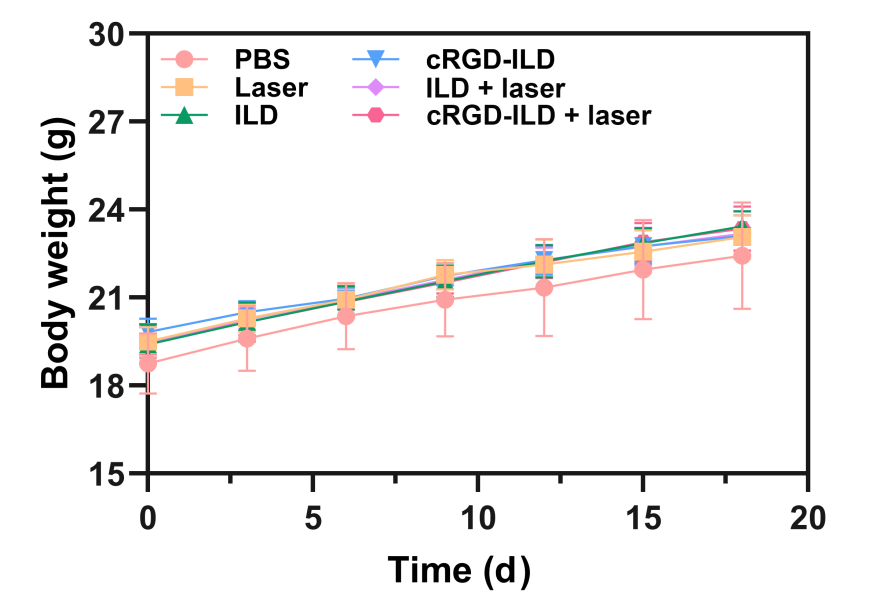


**Figure S22.** Body weight dynamics in EMS model mice during therapeutic intervention. Data are presented as mean ± SD, n = 5 mice per group.


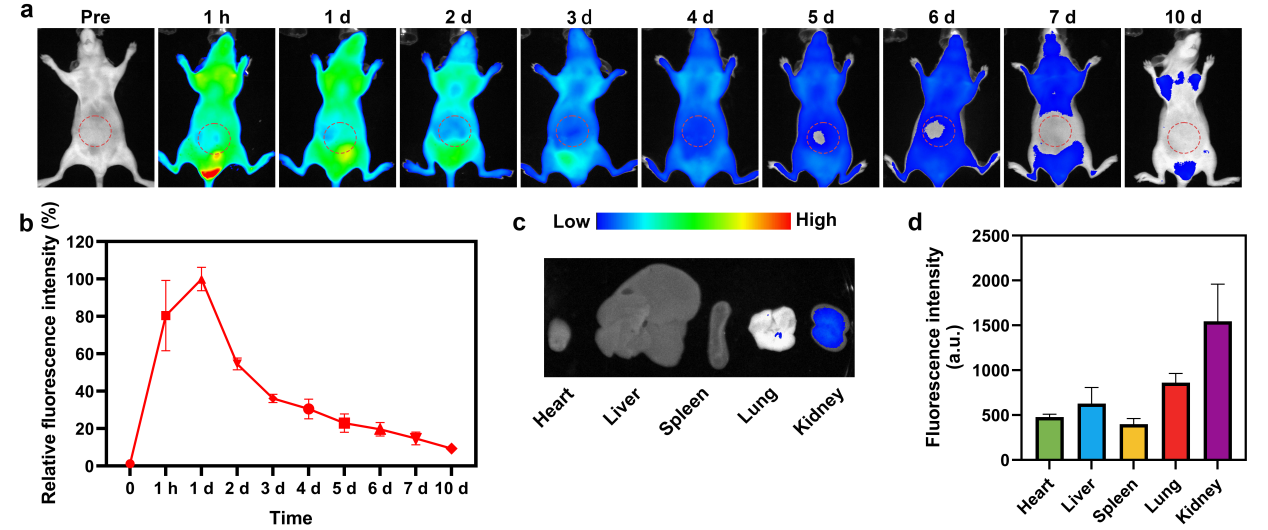


**Figure S23.** *In vivo* fluorescence imaging in healthy BALB/c nude mice. (a) *In vivo* fluorescence images before injection and at various time points after intravenous injection of cRGD-ILD. (b) Time-dependent mean fluorescence intensity within regions of interest (ROIs). Red circles denote ROIs. (c) *Ex vivo* fluorescence imaging of major organs harvested from mice 10 d post-injection in (a). (d) *Ex vivo* fluorescence quantification of major organs (heart, liver, spleen, lung, kidney). Data are presented as mean ± SD, n = 3 mice per group.


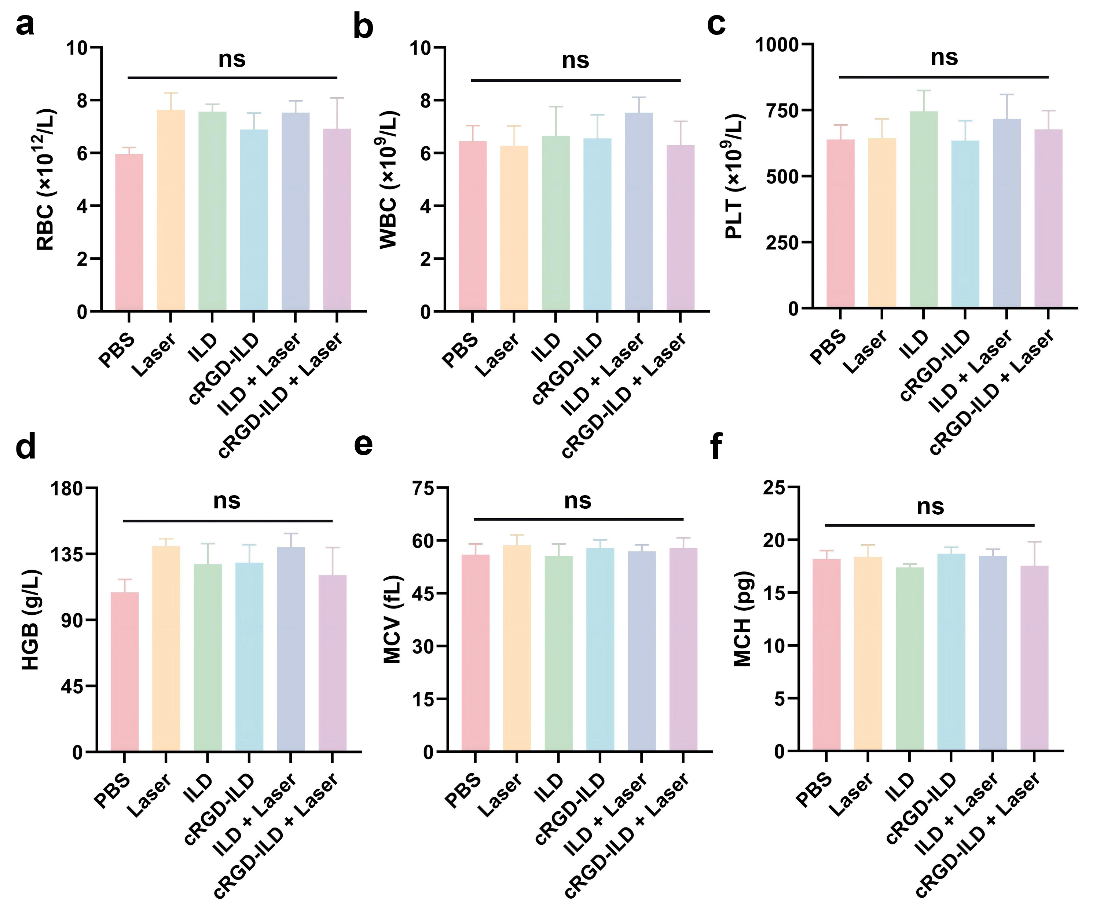


**Figure S24.** Hematological analysis of EMS model rat post-treatment. Peripheral blood analysis of: (a) Red blood cells (RBC), (b) White blood cells (WBC), (c) Platelets (PLT), (d) Hemoglobin (HGB), (e) Mean corpuscular volume (MCV), and (f) Mean corpuscular hemoglobin (MCH). Data are presented as mean ± SD, n = 4 rats per group; ns, not statistically significant, by one-way ANOVA.


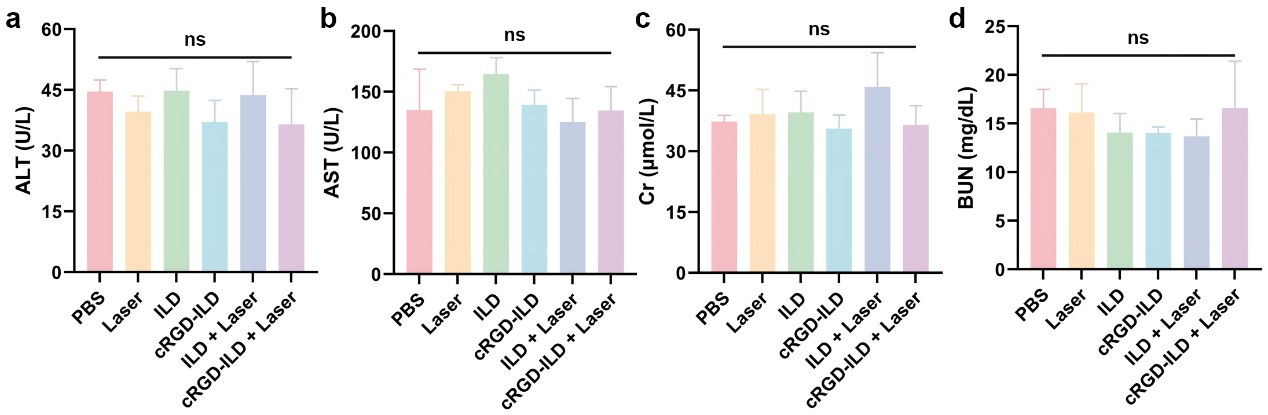


**Figure S25.** Hepatorenal function assessment in EMS model rat post-treatment. Serum biomarker analysis: (a) Alanine aminotransferase (ALT), (b) Aspartate aminotransferase (AST), (c) Creatinine (Cr), and (d) Blood Urea Nitrogen (BUN) in peripheral blood. Data are presented as mean ± SD, n = 4 rats per group; ns, not statistically significant, by one-way ANOVA.


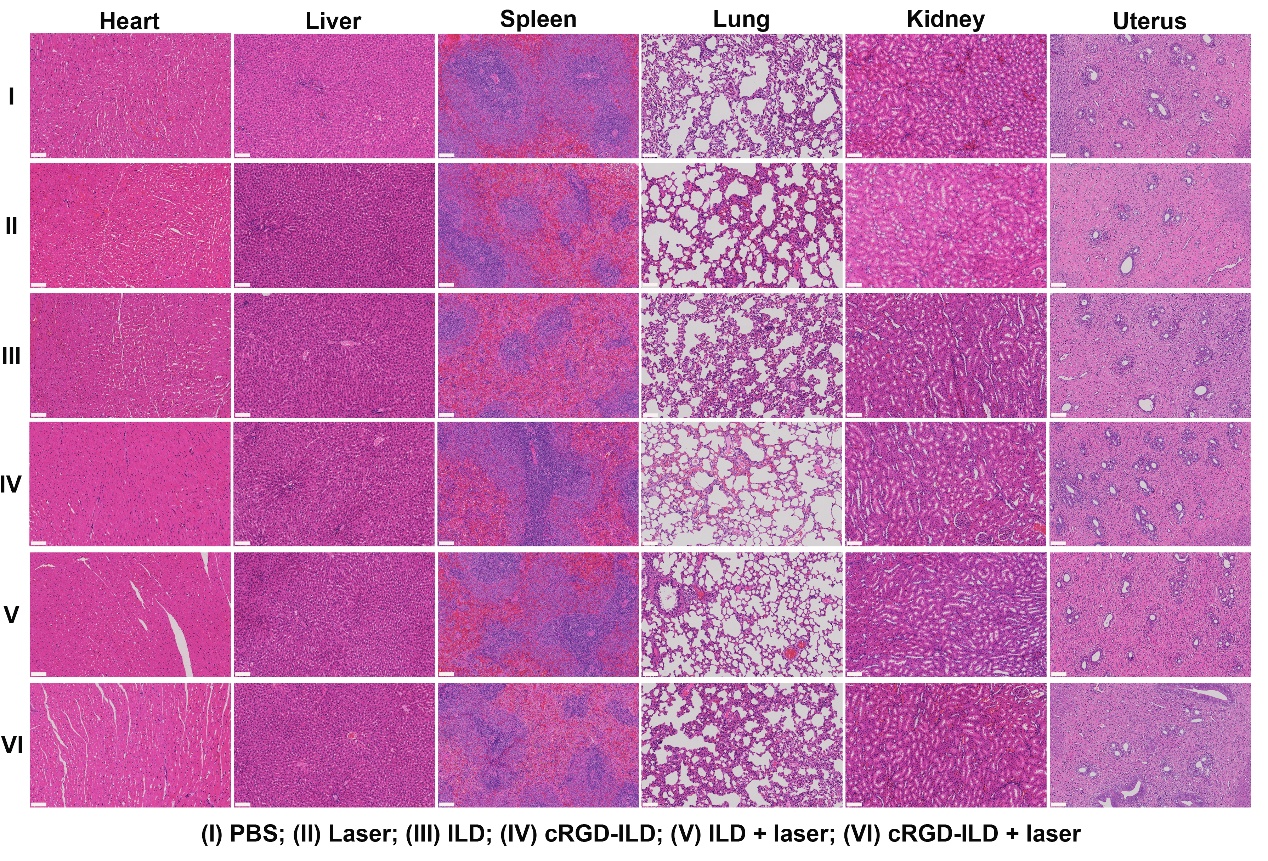


**Figure S26.** H&E staining of major organs and uterus obtained from EMS model rat post-treatment. Scale bar: 100 µm.

## Supplementary Movies

**Movie S1.** Dynamic photoacoustic imaging of the EMS model in nude mice captured at 24 h post-injection of cRGD-ILD. Relatively high photoacoustic signal intensity can be observed in the lesion areas and surrounding vessels.

**Movie S2.** Dynamic photoacoustic imaging of the EMS model in nude mice captured at 24 h post-injection of ILD. The lesion boundaries are indistinct, and the photoacoustic signal is suboptimal around vessels in the lesion periphery.
